# Hydrogel-Controlled Electropolymerization of Polynorepinephrine through Confined Noncovalent Self-Assembly

**DOI:** 10.1021/acs.langmuir.6c04478

**Published:** 2026-10-19

**Authors:** Agata Smułka, Mirosław Sawczak, Łukasz Gaweł, Piotr Okoczuk, Tadeusz Ossowski, Adrian Olejnik

**Affiliations:** † Department of Analytical Chemistry, 49646 University of Gdańsk, Wita Stwosza 63, Gdańsk 80-308, Poland; ‡ Photophysics Department, Institute of Fluid−Flow Machinery, Polish Academy of Sciences, Fiszera 14, Gdańsk 80-231, Poland; § Department of Corrosion and Electrochemistry, Faculty of Chemistry, Gdansk University of Technology, Narutowicza 11/12, Gdansk 80-233, Poland; ∥ Faculty of Applied Physics and Mathematics, Institute of Nanotechnology and Materials Engineering, 49557 Gdańsk University of Technology, Narutowicza 11/12, Gdańsk 80-233, Poland; ⊥ Department of Optoelectronics, Faculty of Electronics, Telecommunications and Electronics, 49557 Gdańsk University of Technology, Narutowicza 11/12, Gdańsk 80-233, Poland

## Abstract

Polymerization of monomers confined within hydrated polymer networks represents a promising strategy for engineering multifunctional, bioinspired interfaces. However, the role of the hydrogel environment in directing polymer growth and the resulting physicochemical properties remains poorly understood. Here, we demonstrate that chitosan hydrogels act as chemically programmed confined reaction environments that govern the electropolymerization of polynorepinephrine (PNOR). The polymer growth inside the matrix leads to hydrogel restructuring and hierarchical noncovalent self-assembly with only minor changes in the chemical composition of both components. The resulting composite exhibits permanently immobilized catechol/quinone redox centers, enhanced broadband optical absorption, and pronounced fluorescence quenching, demonstrating coupled electrochemical and photophysical functionality. Density functional theory reveals that electrostatic interactions are the main factor driving the composite formation with the supportive role of π–cation and hydrogen bonds. Moreover, oxidation of PNOR from the catechol to quinone state substantially modifies the electronic structure responsible for the observed optical response. The results establish hydrogel-controlled, confinement-driven electropolymerization as a general strategy for programming the electrochemical and optical properties of hydrogel interfaces through controlled molecular organization rather than extensive covalent modification.

## Graphical abstract

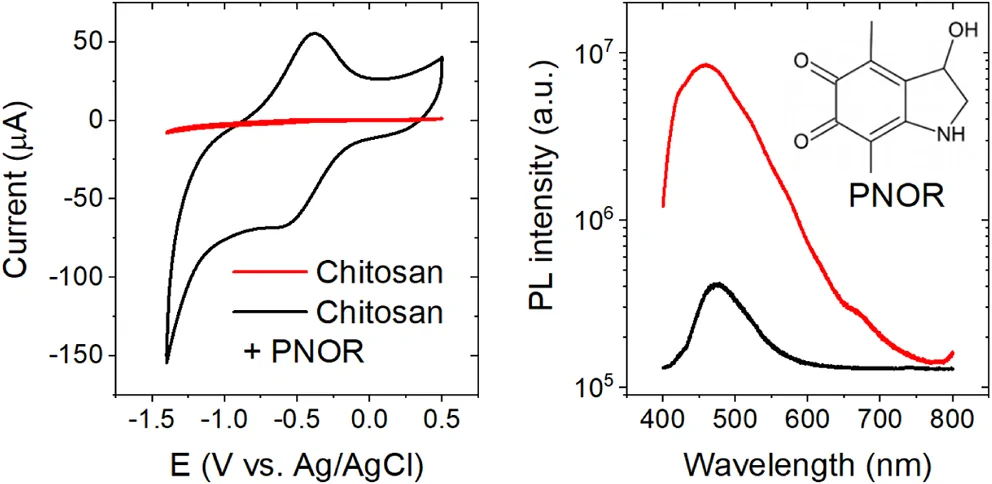

## Introduction

The development of multifunctional hydrogel-based materials integrating structural, electrochemical, and optical functionalities has attracted considerable attention owing to their potential in biosensing, bioelectronics, tissue engineering, and soft electronics.^1^ Incorporation of electroactive polymers into hydrogel matrices further expands their functionality by introducing reversible charge-transfer processes and tunable photophysical properties.^2^ Consequently, such hybrid systems are emerging as promising platforms for electrochemical and bioelectronic interfaces that require simultaneous control of mechanical, electrochemical, and optical characteristics.

Chitosan is a naturally derived polysaccharide obtained by the deacetylation of chitin and characterized by a high density of amino groups that impart excellent film-forming ability and a strong propensity for hydrogel formation. The three-dimensional structure of chitosan hydrogels is primarily stabilized through hydrogen bonding and electrostatic interactions, making their physicochemical properties highly sensitive to parameters such as pH, degree of deacetylation, and ionic strength of the surrounding medium.^3,4^ Beyond the traditional applications in biomedical fields,^5^ chitosan has emerged as an attractive platform for the incorporation of functional components that impart electrochemical, optical, catalytic, or sensing capabilities, enabling the development of multifunctional hybrid materials for bioelectronics and electrochemical interfaces. Representative examples of additives include polyphenolic tannins, which impart antioxidant and photoprotective properties while preserving the structural integrity of the hydrogel,^4^ and polyzwitterions, which improve the mechanical performance, swelling resistance, and hemocompatibility of composite systems intended for biomedical applications.^5^ These studies illustrate that the physicochemical properties of chitosan hydrogels can be effectively tailored through the incorporation of complementary functional components.

Although chitosan is intrinsically electrically insulating, it can efficiently transport protons and hydroxide ions through hydrogen-bonded networks via the Grotthuss hopping mechanism.^6^ This ionic conductivity makes chitosan a promising matrix for the fabrication of electroactive hydrogels, where incorporated redox-active species provide electronic charge-transfer pathways while the hydrogel preserves its hydrated and mechanically compliant nature. Such hybrid materials are increasingly explored as functional interfaces for electrochemical sensing, bioelectronics, and soft electronic devices, where the interplay between ionic transport and reversible redox processes is essential.^7–9^ Nevertheless, in most of the currently adopted systems, chitosan acts primarily as a mechanically supporting or processing matrix, whereas the electrochemical or electronic functionality originates from the incorporated conductive component. Moreover, the concept of chitosan as a confinement medium for reactions is also underexplored.

Bioinspired catechol-derived polymers, particularly polydopamine (PDA) and polynorepinephrine (PNOR), have emerged as attractive functional components for imparting electrochemical activity to otherwise insulating materials. Although the precise molecular structure of these polymers remains a subject of ongoing investigation,^10^ their reversible electrochemical behavior is generally attributed to catechol/quinone redox couples that remain active after polymerization.^11^ Consequently, PDA and PNOR can undergo multiple oxidation states and reversibly exchange electrons, enabling them to function as redox mediators and electroactive interfaces.^11,12^ These properties have led to their application in electrochemical sensors, antifouling coatings, bioelectronic interfaces, and energy-storage materials.^13–19^ Among catechol-derived polymers, PDA has been extensively combined with chitosan to fabricate multifunctional hydrogels and composites. Most reported systems have been developed for biomedical applications, including tissue engineering, wound healing, and drug delivery,^20–22^ while environmental remediation^23^ and sensing platforms^24^ have also been demonstrated. In addition, catechol-functionalized chitosan hydrogels have been shown to exhibit redox-mediated charge transport, highlighting the potential of combining chitosan with catechol chemistry to create electroactive soft materials.^25^ Despite these advances, analogous systems based on PNORs remain largely unexplored, particularly those prepared by electropolymerization. Compared with conventional oxidative polymerization, electropolymerization offers precise control over film thickness, polymer growth kinetics, oxidation state, and spatial localization on conductive substrates, making it especially attractive for the construction of well-defined electroactive hydrogel interfaces.

Compared with PDA, PNOR has received considerably less attention despite possessing an additional β-hydroxyl group that may influence intermolecular interactions, polymer growth, and the resulting physicochemical properties. Consequently, the mechanisms governing PNOR electropolymerization and the relationship between its molecular structure, electrochemical activity, optical properties, and electronic structure remain significantly less understood. In the context of recent progress in confined polymerization, it remains unclear how the hydrogel environment influences PNOR formation and how these structural changes govern the properties of the resulting composite.^26^ Addressing these relationships is essential for the rational design of multifunctional electroactive hydrogel interfaces for sensing and bioelectronic applications.

In this work, we present a study of how a confined hydrogel environment of the chitosan directs the formation and properties of a PNOR electropolymer. We address this knowledge gap by investigating the electropolymerization of PNOR within a preformed chitosan hydrogel deposited on a glassy carbon electrode (GCE) with coupled electrochemical and optical functionalities. The influence of hydrogel preparation pH and polymerization conditions on the morphology, electrochemical behavior, optical properties, and surface chemistry of the resulting material is systematically examined. Particular attention is devoted to establishing structure–property relationships by correlating surface morphology, permanent redox activity, optical absorption, fluorescence, and electronic structure. Density functional theory calculations are employed to rationalize the experimentally observed optical behavior by elucidating the influence of chitosan cross-linking and PNOR oxidation state on the electronic bandgap. The obtained results provide new insight into the design of electroactive catechol-based hydrogels and establish design principles for electroactive catechol-based hydrogel interfaces. To the best of our knowledge, this study represents the first systematic investigation of PNOR electropolymerization within a chitosan hydrogel matrix and establishes fundamental structure–property relationships governing the electrochemical and photophysical behavior of the resulting hybrid interface.

## Experimental Section

### Preparation of Electrodes Modified with Chitosan Hydrogels Containing PNOR

Information regarding materials and reagents can be found in the Supporting Information. The chitosan hydrogel was prepared according to the previously described procedure.^27,28^ Appropriate amounts of chitosan were suspended in a 1% acetic acid solution and then mechanically stirred for 24 h. Next, suitable amounts of glutaraldehyde were added and mixed again for 1 h. To some of the hydrogels obtained, 0.2 M NaOH was added to achieve the desired pH, and they were left to mix for another 2 h. The materials obtained were filtered to remove any unsolved reagents. The prepared hydrogels were stored in a refrigerator at 4 °C. Glassy carbon electrodes with a diameter of 3.0 mm were prepared by polishing with 0.3 mm Al_2_O_3_ powder on a Buehler set. They were then washed with deionized water and dried with a nitrogen stream. 5 μL of chitosan hydrogel was applied to the electrode surfaces, and then dried in a nitrogen stream for 30 min. Electropolymerization of norepinephrine (NOR) was performed by conducting 100 cycles of voltammetry at a scanning rate of 50 mV/s in the potential range from −0.7 to +1.2 V relative to Ag|AgCl. The solution contained Tris buffer with an appropriate pH and 10 mM norepinephrine. Before starting the electropolymerization, argon gas was passed through the solution for 20 min. All procedures were performed at room temperature. The pH values of the above-mentioned solutions were monitored using a pH electrode.

### Physicochemical Measurements

Details regarding the experimental setups for atomic force microscopy (AFM), scanning electron microscopy (SEM), X-ray photoelectron spectroscopy (XPS), profilometry, optical microscopy, Raman spectroscopy, UV–vis spectrophotometry, photoluminescence (PL), and water contact angle (WCA) measurements are presented in the Supporting Information.

### Electrochemical Measurements

All electrochemical measurements were performed using a Methron Autolab M204 potentiostat/galvanostat equipped with an FRA32 M electrochemical impedance spectroscopy module running a Nova 2.15 operating system. The experiments were conducted in a three-electrode system, where the working electrode was a hydrogel-modified GCE, the reference electrode was Ag/AgCl (0.1 M KCl), and platinum served as the counter electrode. Investigations were performed with redox probes consisting of 1 mM solutions of K_3_[Fe­(CN)_6_], K_4_Fe­(CN)_6_, and C_12_H_14_FeO_2_, dissolved in 0.1 M KCl or PBS as the main electrolyte or in pristine buffer solutions.

### Quantum Chemistry Simulations

All density functional theory (DFT) and associated COSMO-DFT solvation simulations were performed using the QuantumATK v2025.09 software package from Synopsys.^29^ All of the molecular structures were created using the Builder tool of the ATK package. Further details can be found in the Supporting Information.

## Results and Discussion

### Confined Electropolymerization of NOR in Chitosan Hydrogels

To investigate how the hydrogel environment governs PNOR electropolymerization, we prepared chitosan hydrogels with different internal protonation states by varying the synthesis pH (Scheme 1). Because the protonation state of chitosan is largely retained after hydrogel formation, these materials provide confined reaction environments with distinct electrostatic characteristics for PNOR growth.^27^ In accordance with the described procedure, hydrogels with pH 3 (Cs3) and pH 7 (Cs7) were synthesized. Depending on the chitosan synthesis pH, the ratio of protonated to deprotonated amine groups is expected to vary. Based on our observations, the internal pH of chitosan remains stable regardless of the external electrolyte pH. Throughout this work, the chitosan hydrogel is considered to be a confined electropolymerization medium in which restricted diffusion, local proton concentration, and electrostatic interactions collectively influence PNOR formation.

The electrodes coated with hydrogel were subjected to electropolymerization in the presence of 10 mM norepinephrine in Tris buffers by cyclic voltammetry (CV) in a potential range from −0.7 to 1.2 V. The choice of parameters was dictated by the well-established procedures for PDA and its analogues electropolymerizations.^30^ Instead of the PDA, NOR was chosen because it was shown that PNOR coatings are more uniform compared to the PDA.^31^ On a pure GCE surface (Figure 1a and Figure S2), a series of oxidation and reduction peaks are present due to catechol-quinone redox reactions. Overall, a broad and irreversible anodic oxidation wave is a signature of the polymerization. With subsequent CV scans, the current decreases in the whole potential window and peak-to-peak distances are larger. An additional oxidation peak appears in the mildly cathodic region during the second CV cycle, confirming the formation of the PNOR electropolymer. These PNOR-specific redox peaks in the cathodic area emerge during the second CV cycle consistently for both free and confined electropolymerizations.

**Figure 1:**
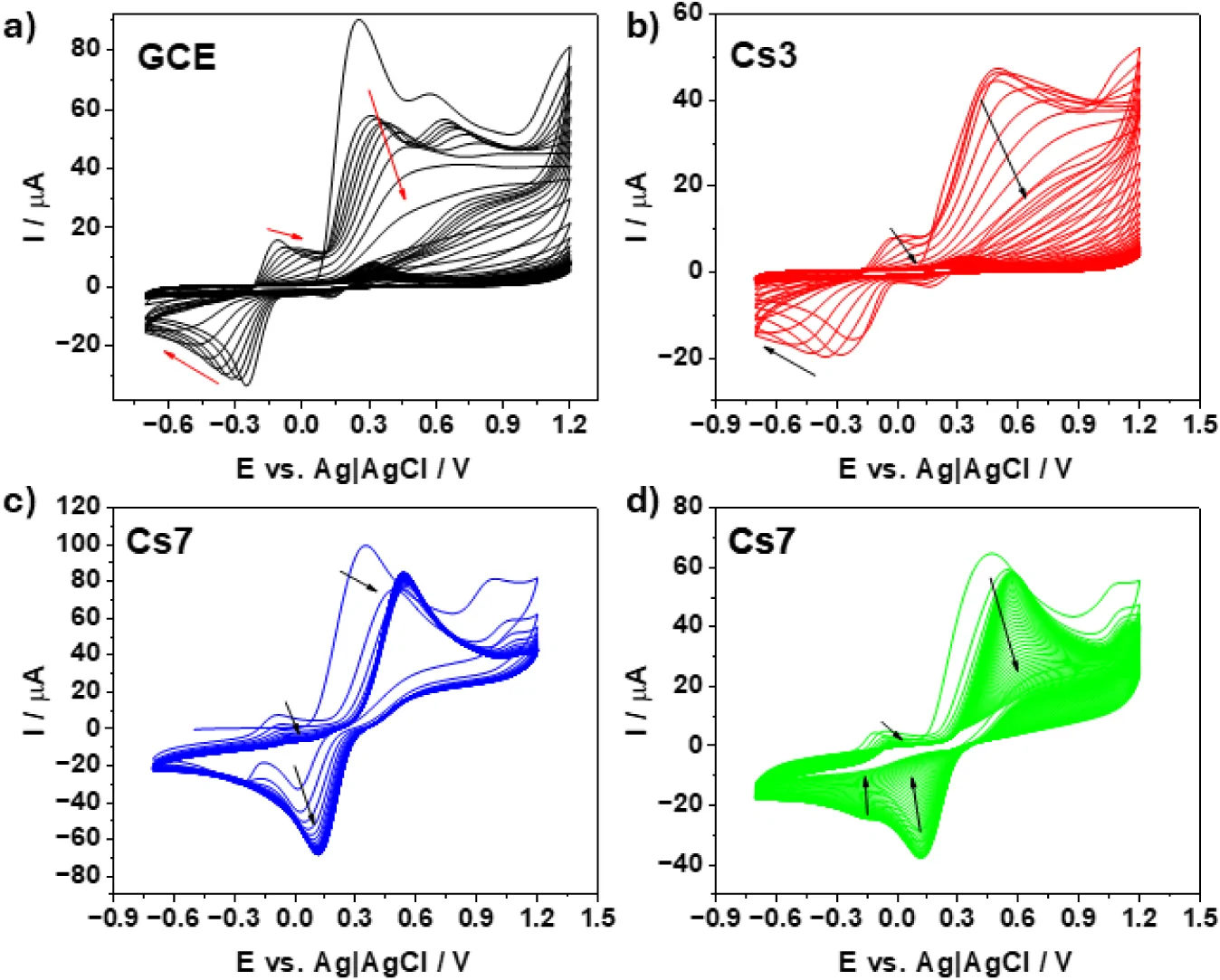
NOR electropolymerization on a) pristine GCE, b) confined in Cs3 at pH = 7.4, c) confined in Cs7 at pH = 6.0, and d) confined at pH = 7.4.

The CV shape for NOR electropolymerization on the surface of GCE-Cs3 at an external pH of 7.4 strongly resembles that of the pristine GCE with slightly shifted redox peaks (Figure 1b) due to the lower internal pH of the Cs3 chitosan compared to the external one. However, the current values are lower, but they do not disappear so rapidly in subsequent scans, indicating slower kinetics of polymerization in the confined environment. The progressive shift of the cathodic process indicates the continuous evolution of PNOR during film growth. However, the use of the Cs7 matrix leads to significant changes in the course of the polymerization due to confined character (Figure 1c and d). In the anodic region, there is a single redox peak instead of a quasi-plateau character. Additionally, there are no significant shifts in potentials during cyclization and only a gradual decrease in current values. In contrast to Cs3, peaks do not fully disappear after cyclization, indicating permanent conferring of the PNOR redox couples. Therefore, confined electropolymerization inside Cs7 is fundamentally different compared to Cs3 even if the external electrolyte pH is the same.

Oxidation and reduction of dopamine are highly pH-dependent due to the proton-coupled electron transfer (PCET) mechanism; hence, similar dependencies can be expected for NOR. If the polymerization was confined inside Cs7 with external pH reduced to pH 6, there is no progressive decrease in current magnitudes in subsequent CV cycles, suggesting that no effective polymerization process is occurring. If the external pH is too acidic, the polymerization will not be triggered even if the internal pH of the chitosan confinement zone is greater because the protonated monomer exists in an external medium. Therefore, PNOR electropolymerization is governed by two coupled protonation equilibria: the internal protonation state of the hydrogel, which defines the confined reaction environment, and the protonation state of NOR in the external electrolyte, which controls the influx of the monomer into the hydrogel and the kinetics of oxidative polymerization.

The pathway of NOR electropolymerization is expected to be similar in essence to that of PDA. Briefly, after catechol/quinone proton-coupled electron transfers, monomers undergo Michael cyclization and further tautomerizations (Scheme S1). Subsequently, monomers on different oxidation states and in various tautomeric forms undergo aryl–aryl coupling to form chains, which then undergo chemical and physical cross-linking (Scheme S2). The mechanism of the electropolymerization confined inside hydrogels with variable pH is expected to differ from that of pure GCE for two reasons. First, the crucial chemical step in PNOR formation—Michael cyclization—is strongly pH-dependent.^32^ Although cyclization occurs between pH 4 and 9, it seems most effective in the slightly alkaline range of 7.5–8.5. Second, both chitosan and NOR monomers exhibit different formal charges depending on the pH. NOR molecule has three p*K*_a_ values associated with amine protonation (ca. p*K*_a_ = 9) and catechol protonations (ca. p*K*_a_ = 10 and 12) (Scheme S3). In acidic to neutral pH, NOR is expected to be in the cationic form, with some contribution of neutral and zwitterionic forms. On the other hand, chitosan is expected to undergo amine deprotonation at ca. pH 6/7. Those differences in charge lead to strong electrostatic (ionic) interactions, which significantly impact the electropolymerization process. Increasing the internal hydrogel pH stabilizes the PNOR redox response, suppresses rapid passivation of the electrode, and promotes the formation of electrochemically persistent catechol/quinone functionalities. Therefore, the chitosan hydrogel does not merely serve as a passive support for PNOR deposition. Instead, its internal protonation state fundamentally alters the electropolymerization pathway by both catalytic effects on subsequent steps and the milieu of the electrostatic interactions.

**Scheme 1:**
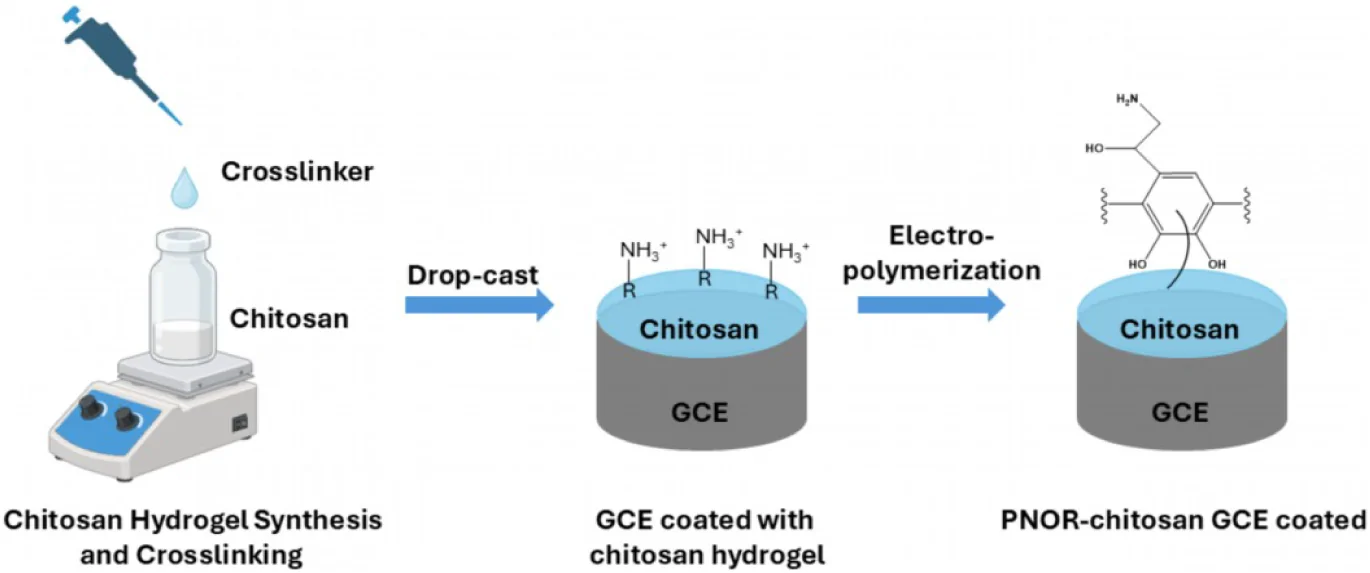
Electrode Cs-PNOR Preparation Procedures

It is known that the oxidation and reduction of dopamine are highly pH-dependent; hence, similar dependencies can be expected for norepinephrine. Figure S4a–c shows the electropolymerization curves of norepinephrine on a Cs7 coating in Tris buffer at pH 6, 7.4, and 8. In the case of the curve recorded at pH 6, an oxidation peak is observed at approximately 350 mV, which shifts to approximately 540 mV, and a signal at approximately 970 mV disappears in subsequent CV cycles. During the second run, a signal also appears at −90 mV. The visible reduction peak at approximately 16 mV shifts to 115 mV, while the signal at −270 mV disappears by the third cycle. The absence of a clear decrease in oxidation and reduction signals in subsequent CV cycles suggests that no effective polymerization process is occurring, indicating that the medium with a pH of 6 is too acidic for this process to take place. There is also the possibility of a conductive polymer forming, or a soluble product, which may also manifest as an absence of a redox signal. In contrast, at pH 8, oxidation peaks are visible at approximately 360 mV (shifting to about 530 mV), at around 1 V, and, starting from the second cycle, at roughly −120 mV. A reduction peak is also observed at approximately 85 mV, as well as a signal at approximately −220 mV that fades after the third cycle. The clearly decreasing peak values are characteristic of the electropolymerization process and indicate the accumulation of the reaction product in the near-electrode layer, leading to the gradual blocking of the electrode surface.

We have also performed stability and repeatability testing of the ITO–chitosan–PNOR systems (Figure S3). Three independent samples were prepared according to the procedure described in the paper for Cs3 chitosan and electropolymerization of 10 mM PNOR at pH 7.4 in 1× Tris buffer. All three samples were tested by CVs in a neutral electrolyte (1× Tris). Additionally, to make the stability test more demanding, measurements were performed over a broad potential window (from −1.0 V to +1.5 V limited by the hydrogen/oxygen evolution reaction) and a broad space of scan rates (from 100 mV/s to the fast-scan voltammetry limit of 10 V/s).

Three redox couple signatures at potentials −250 mV, +500 mV, and +800 mV are observed similarly for all three samples, although the resolution of particular redox peaks varies. Nonetheless, each sample underwent 1500 cycles, with no significant change in the shape of the voltammogram. For sample 3, the cycling was performed even further to 10,000 cycles. Redox peak signatures are still well resolved even after 5000 cycles, while after 10,000, their resolution clearly becomes poorer.

To rationalize the experimentally observed pH dependencies, DFT-COSMO calculations were performed to evaluate how protonation influences intermolecular interactions. Charge density maps in Figure S6 show that the cationic form of NOR is a strong H-bond donor, the anionic form is a strong H-bond acceptor, and the zwitterionic form has both capabilities. No major differences were observed between catechol/quinone variants (Table S2 and Figure S8). Therefore, it is the pH rather than the oxidation state that determines the H-bond character. The calculated decrease in solvation energy with increasing chain length (Table S3) suggests that oligomer growth is accompanied by progressive loss of solubility, eventually driving precipitation of PNOR within the hydrogel network after exceeding 12 monomers in the chain. This provides a molecular-level explanation for the formation of stable surface-confined polymer films observed experimentally and their alteration due to confinement inside the chitosan.

### Restructuring of Hydrogel Morphology after Electropolymerization

Because electropolymerization occurs within the confined hydrogel network, the initial microstructure of the chitosan matrix is expected to strongly influence the monomer diffusion, polymer nucleation, and subsequent PNOR growth. SEM and optical microscopy were therefore employed to determine how the hydrogel preparation pH modified the confined reaction environment. Cs3 and Cs7 hydrogels exhibit drastic changes in the morphology due to the different growth mechanisms. SEM images (Figure 2) reveal a smooth and consistent morphology of the Cs3 hydrogel on the whole electrode surface, whereas the Cs7 hydrogel exhibits a fibrous structure. These fibers are regular at the phase boundary, which forms during the drying of the hydrogel. In the central part of the sample, however, fibers become more irregular with a marked increase in apparent surface area was observed compared to Cs3.

**Figure 2:**
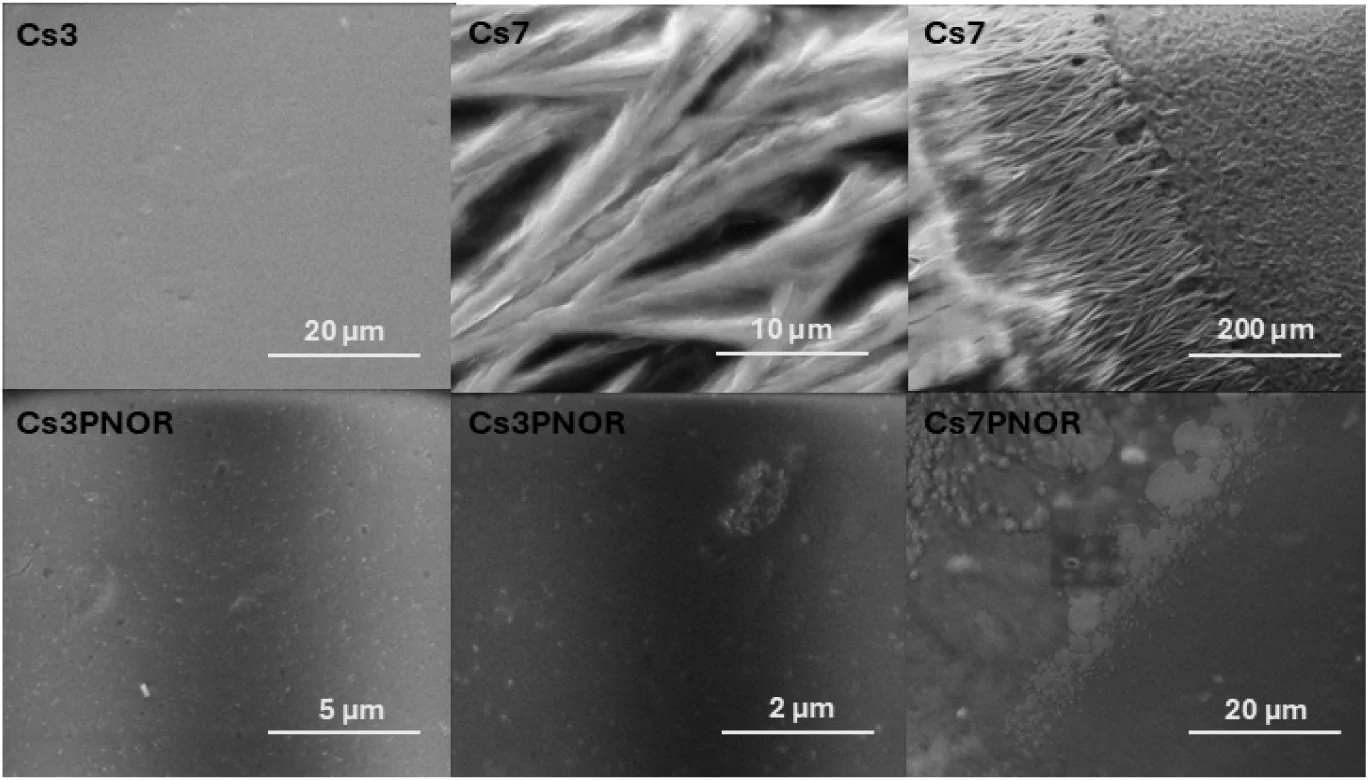
SEM images of chitosan matrices and chitosan–PNOR interfaces.

Fibrous morphologies are commonly obtained for chitosan through electrospinning or mechanically directed self-assembly.^4,33–40^ Their spontaneous appearance in drop-cast hydrogels is therefore unexpected and suggests that the higher synthesis pH fundamentally alters the gelation and drying processes. We attribute this behavior to partial deprotonation of chitosan, accompanied by replacement of acetate counterions with hydroxide ions, which promotes increased crystallinity and reduced polymer solubility during drying. The pronounced differences in the microstructure imply that PNOR electropolymerization occurs within fundamentally different confined environments in Cs3 and Cs7 hydrogels. Consequently, not only the local proton concentration but also the available internal surface area, diffusion pathways, and nucleation sites for polymer growth are expected to be different substantially.

Electropolymerization of PNOR induced pronounced but hydrogel-dependent structural reorganization. Although Cs3 underwent a general increase in surface roughness, PNOR formation within Cs7 preferentially modified the preexisting fibrous regions, producing highly porous domains, while simultaneously smoothing the central areas of the hydrogel. Optical microscopy images (Figure S1) indicate uniform coating of the electrode surfaces with both of the studied hydrogels. A smoother and more homogeneous structure with no visible gaps or agglomerates is reported for pristine Cs3. At the same time, a heterogeneous structure of the pristine Cs7 hydrogel layer and its PNOR modification was observed, which is consistent with the SEM analysis results. These contrasting responses indicate that PNOR growth is governed by the local microstructure of the confined hydrogel rather than occurs uniformly throughout the film.

The experimentally observed morphology is consistent with the calculated solvation energies. DFT-COSMO predicts a substantial decrease in chitosan solvation upon deprotonation (−2.92 eV to −0.61 eV), indicating a strong reduction in polymer–solvent interactions under alkaline conditions. Reduced solvation favors chain association and partial ordering during drying, providing a molecular-level explanation for the emergence of the fibrous microstructure. Together, the microscopy and DFT indicate that the hydrogel microstructure does not simply support PNOR deposition but actively defines the reaction environment in which electropolymerization proceeds.

PNOR electropolymerization induces the structural reorganization of the confined hydrogel network. Thus, simultaneous changes in the surface roughness, wettability, and film thickness are expected . AFM, water contact angle, and profilometry measurements were therefore performed to evaluate how the PNOR formation modifies the hydrogel architecture.

The AFM results reveal two fundamentally different responses to PNOR formation (Figure 3 and Table 1). In Cs3, electropolymerization produces only a modest decrease in roughness, consistent with the deposition of a relatively uniform PNOR layer on an already compact hydrogel surface. Conversely, Cs7 undergoes a pronounced decrease in roughness, indicating that PNOR formation is accompanied by a substantial reorganization of the initially heterogeneous hydrogel network.

**Figure 3:**
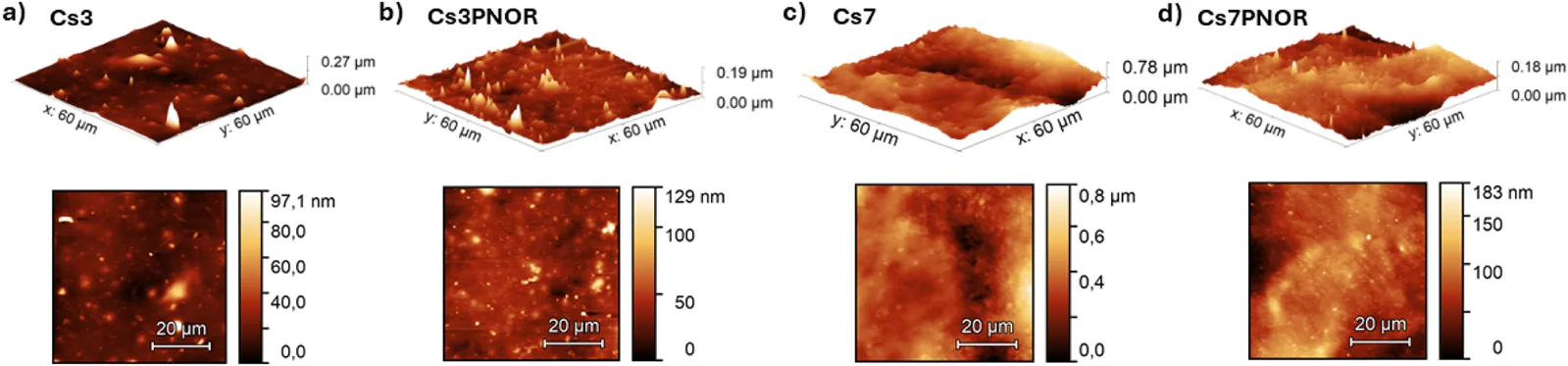
AFM micrographs of GCE layers coated with layers (Cs3, Cs3-PNOR, Cs7, Cs7-PNOR).

**Table 1:** Water Contact Angle, Surface Roughness Parameters, and Layer Thicknesses from Profilometry

	WCA [°]	SFE [mN/m]	SFE-disperse [mN/m]	SFE-polar [mN/m]	Mean Ra [nm]	Mean Rq [nm]	Layer thickness [μm]
Cs3-PNOR	72.26	45.44	38.95	6.49	8.00	12.21	2.5
Cs7-PNOR	60.78	46.64	37.32	9.32	18.72	23.82	0.5
Cs3	68.91 ^27^	52.04 ^27^	31.67 ^27^	19.37 ^27^	11.78 ^27^	-	3
Cs7	50.87 ^27^	49.21 ^27^	31.07 ^27^	18.13 ^27^	111.24 ^27^	-	5

The wettability measurements support this interpretation. Incorporation of PNOR decreases the hydrophilicity of both hydrogels, with the effect being substantially more pronounced for Cs7. The simultaneous increase in water contact angle and reduction of the polar surface energy component indicate that PNOR formation reduces the accessibility of hydrophilic groups at the interface, consistent with partial collapse or densification of the hydrogel network observed in SEM in Figure 2. Although Cs3 exhibits only a modest reduction in thickness (≈3.0 to 2.5 μm), Cs7 contracts by nearly an order of magnitude (≈5.0 to 0.5 μm). Profilometry provides direct evidence that PNOR formation is accompanied by large-scale contraction of the hydrogel matrix.

Taken together, the AFM, wettability, and profilometry results indicate that PNOR formation is accompanied by the pH-dependent restructuring of the hydrogel network. Although the highly protonated Cs3 hydrogel primarily serves as a passive scaffold for PNOR deposition, the partially deprotonated Cs7 hydrogel undergoes pronounced densification associated with water expulsion and the rearrangement of the polymer network. These observations further support the concept that the hydrogel actively participates in the electropolymerization process rather than serving as an inert support.

The experimentally observed contraction of Cs7 is consistent with the DFT-predicted decrease in chitosan solvation upon deprotonation (Figure S7). Reduced affinity toward water lowers the energetic penalty associated with hydrogel collapse, thereby facilitating densification of the polymer network during PNOR formation. Rather than simply coating the hydrogel surface, PNOR electropolymerization restructures the hydrogel itself with the extent of this reorganization being dictated by the protonation state of the chitosan matrix.

### DFT-COSMO Simulations of Intra- and Intermolecular Interactions within PNOR–Chitosan

To identify the molecular interactions responsible for the formation and stability of the PNOR–chitosan composite, DFT-COSMO calculations were performed for the three most probable intermolecular interaction mechanisms: electrostatic attraction/repulsion, π–cation interactions, and hydrogen bonding in different configurations (Schemes S4–S6). The calculations reveal a clear hierarchy of intermolecular interactions for both PNOR and PNOR–chitosan associates. Electrostatic attraction is energetically dominant, whereas hydrogen bonding and π–cation interactions are considerably weaker, as shown in Table 2. Detailed values of interaction energies corresponding to, e.g., different types of intra- and intermolecular hydrogen bonding can be found in Tables S7–S9.

**Table 2:** Comparison of DFT-COSMO-Calculated Interaction Energies between NOR–NOR and NOR–Chitosan Models for Various Types of Noncovalent Association Patterns

	π–cation	Strongest H-bonds	Electrostatic (ionic association)
NOR–NOR	–0.038	–0.264	–0.704
NOR–chitosan	–0.270	–0.158	–0.630

The calculations indicate that change of the pH equilibria and thus change in electrostatic interactions act as a molecular “switch” controlling composite formation. Attractive electrostatic (ionic) interactions become possible only when PNOR adopts a zwitterionic or anionic form, while chitosan retains a sufficient population of protonated amino groups. Under more acidic conditions, electrostatic repulsion between protonated PNOR and protonated chitosan prevents efficient incorporation of PNOR into the hydrogel (compare with poor electropolymerization progress at pH 6, Figure 1c). Conversely, excessively alkaline conditions would generate predominantly anionic PNOR species, introducing electrostatic repulsion between polymer chains. This prediction is fully consistent with the electrochemical experiments, in which efficient electropolymerization was observed only within a narrow pH window (approximately pH 7–8), indicating that successful composite formation requires a balance between the protonation states of both components.

Although substantially weaker than electrostatic interactions, π–cation interactions provide an additional stabilization mechanism. The calculations indicate that PNOR–chitosan π–cation interactions are considerably stronger than the corresponding PNOR–PNOR interactions, suggesting that aromatic PNOR units preferentially interact with protonated chitosan rather than with one another (Table 2). Hydrogen bonding plays a different role. Strong hydrogen-bonding networks are predicted within both PNOR and chitosan individually, contributing to the structural integrity of each component. In contrast, hydrogen bonds formed directly between PNOR and chitosan are consistently weaker, indicating that they are unlikely to be the primary driving force responsible for composite formation.

Collectively, the DFT results suggest that the PNOR–chitosan composite is assembled through a hierarchical interaction mechanism. Electrostatic attraction governs the initial incorporation of PNOR into the hydrogel, π–cation interactions reinforce the association between aromatic PNOR units and protonated chitosan, while hydrogen bonding primarily stabilizes the internal structures of the individual polymer networks. This hierarchical self-assembly provides a molecular-level explanation for the pronounced pH dependence observed experimentally during confined electropolymerization. Direct covalent coupling between oxidized PNOR species and nucleophilic amino groups of chitosan cannot be completely excluded.^41^ However, the XPS analysis presented below reveals only minor chemical changes, suggesting that noncovalent interactions dominate the composite formation process.

### Chemical Structure of the Noncovalently Self-Assembled Interfaces

To determine whether the pronounced differences in morphology and electrochemical behavior originate from changes in surface chemistry or from differences in the organization of the hydrogel network, XPS analysis was performed for pristine and PNOR-modified chitosan hydrogels (Figure 4 and Table S1).

**Figure 4:**
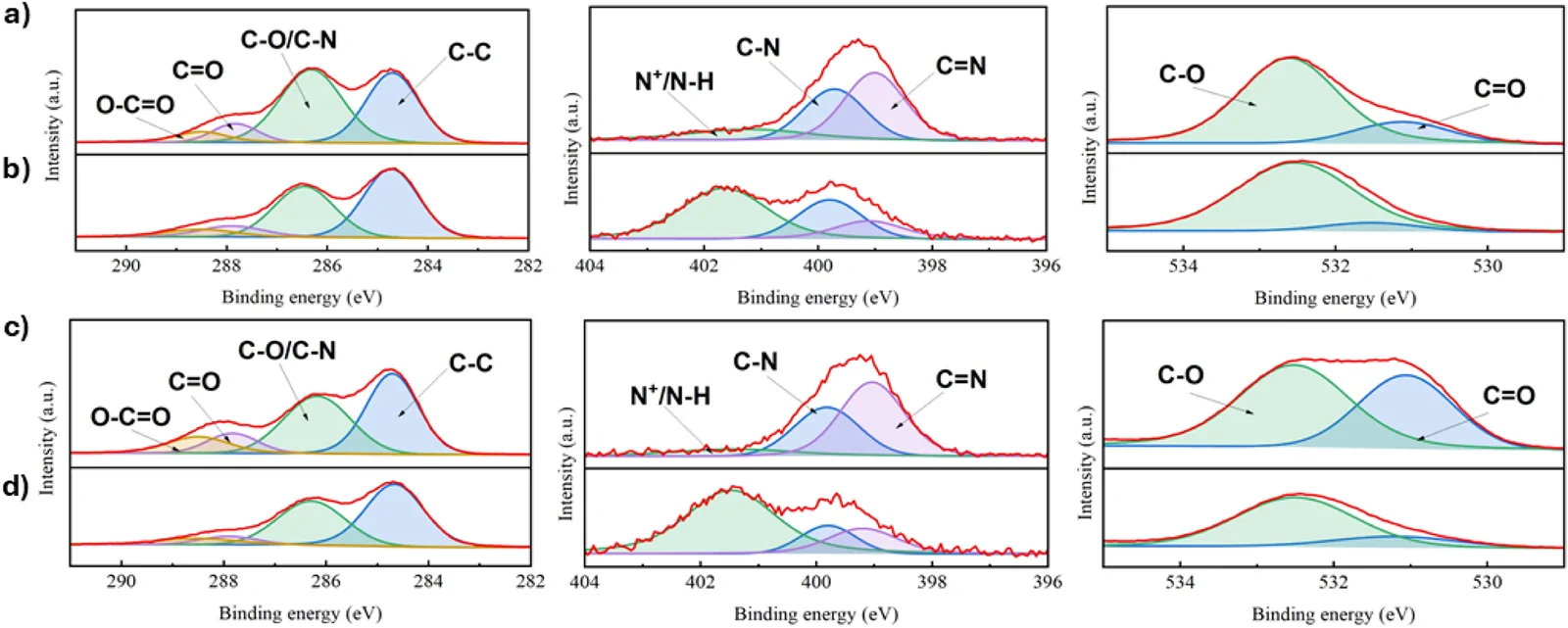
High-resolution XPS spectra of a) Cs3; b) Cs3-PNOR; c) Cs7; and d) Cs7-PNOR hydrogel, performed for C 1s, O 1s, and N 1s regions.

Both PNOR-modified samples exhibit clear spectroscopic signatures of successful polymer incorporation, especially for the N spectrum. However, only relatively minor differences are observed between Cs3-PNOR and Cs7-PNOR, despite their markedly different electrochemical and morphological characteristics.

The increase in the C–C contribution together with the higher fraction of protonated/oxidized nitrogen species confirms the successful incorporation of PNOR into the chitosan matrix. At the same time, only modest variations are observed in the oxygen-containing functional groups, indicating that electropolymerization does not substantially alter the overall surface chemistry of the hydrogel.

The relatively small chemical differences revealed by XPS contrast sharply with the pronounced changes observed in the morphology, roughness, wettability, and electrochemical behavior. Together with DFT calculations, these observations suggest that the distinct properties of Cs3-PNOR and Cs7-PNOR originate primarily from differences in noncovalent intermolecular interactions and hydrogel organization rather than from the formation of substantially different chemical structures.

In the O 1s spectra, the C–O content increased by 3–5%, reflecting the reorganization of hydrogen bonds following modification, with stable C=O groups (change <2%). These surface changes are consistent with the effects of glutaraldehyde cross-linking and pH modification in chitosan hydrogels. Several factors may account for the high C=O signals, including solvent residues (acetic acid), aldehydes, or nondeacetylated chitosan moieties. The lack of increase after polymerization may result from conversion to amines due to interaction with the chitosan chain.

Perhaps the most significant outcome of XPS analysis is not the presence of new chemical functionalities, but rather their absence. Despite the pronounced differences in morphology, electrochemical response, and optical properties (as discussed further), the surface chemical compositions of Cs3-PNOR and Cs7-PNOR remain remarkably similar. This observation indicates that the multifunctional behavior of the composite is governed predominantly by differences in molecular organization and noncovalent interactions rather than by extensive covalent modification of the chitosan network.

### Optical Absorption and Fluorescence Quenching in PNOR–Chitosan Composites

The optical properties of the composite provide an indirect probe of how confined electropolymerization modifies its electronic structure. Electropolymerization of PNOR produces a pronounced increase in optical absorption across the entire UV–vis range, irrespective of the hydrogel preparation pH (Figure 5a). Such broadband absorption is characteristic of melanin-like polymers and indicates the formation of a heterogeneous electronic structure containing multiple optically active states.^42–45^

**Figure 5:**
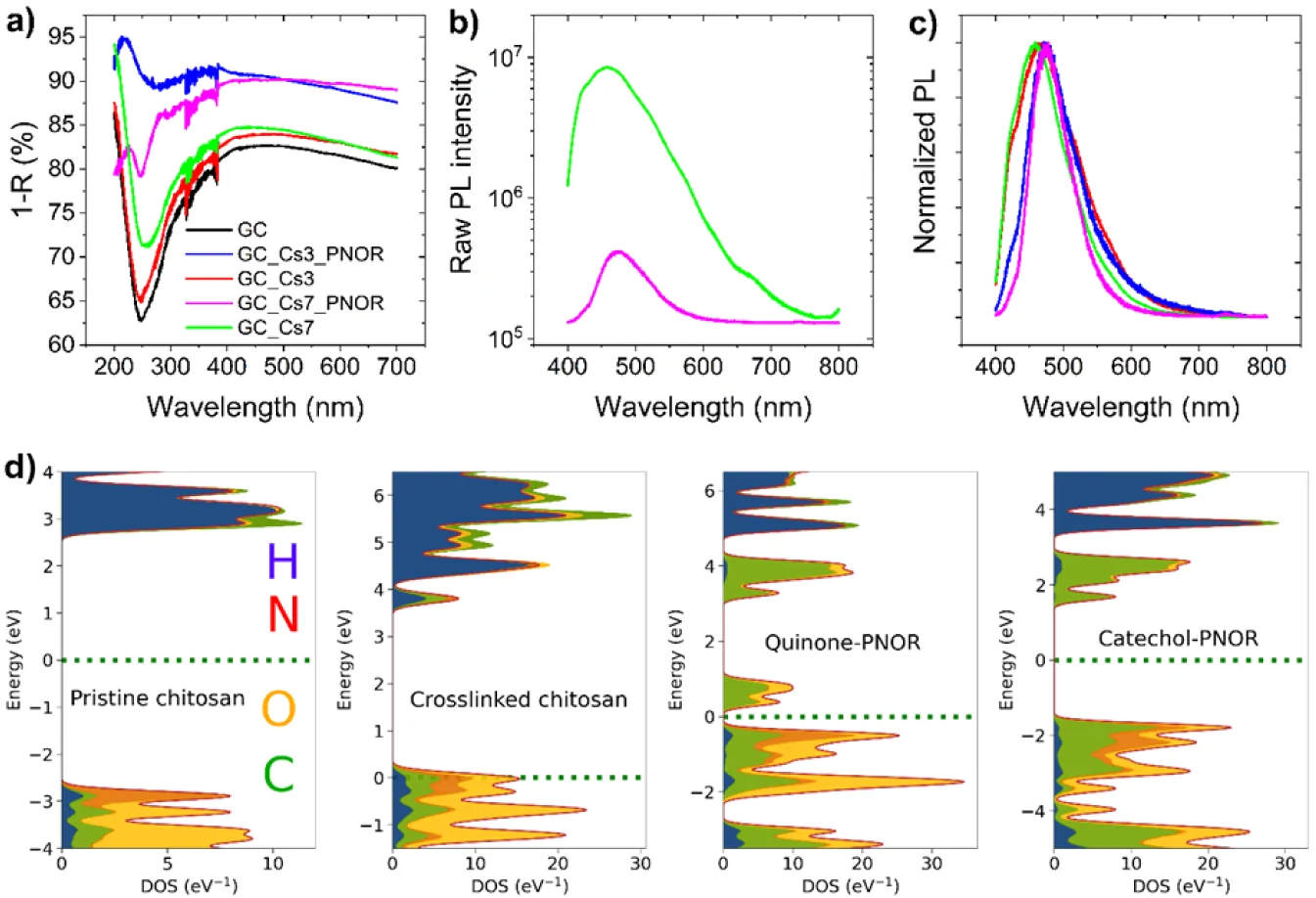
Top row: Optical properties of PNOR–chitosan interfaces: a) UV–vis absorption spectra, b) raw PL spectra of pristine and modified Cs7 hydrogel, c) normalized PL spectra. d) DFT-COSMO-derived, atom-projected DOS spectra of pristine chitosan trimer, cross-linked chitosan, and two variants of PNOR models: quinone and catechol trimers. Atomistic structures are given in Figures S11 and S12.

To rationalize this broadband optical response, DFT calculations were performed for PNOR models representing different oxidation states. Oxidation of catechol units to quinones dramatically reduces the electronic bandgap (4.1 eV → 1.7 eV) and shifts the Fermi level downward (Figure 5d and Table S4). The same property is observed for both monomers and trimer models. Additionally, increasing polymer length is another factor reducing the bandgap (Figures S9–S10 and Table S5) Consequently, the coexistence of oligomers with different oxidation states and chain lengths naturally produces the broad distribution of electronic transitions responsible for the experimentally observed absorption.

Pristine chitosan exhibits strong PL signals under 365 nm excitation regardless of the preparation pH (Figure 5b). DFT-COSMO calculations indicate that glutaraldehyde cross-linking substantially modifies the electronic structure of chitosan by reducing the HOMO–LUMO gap to ca. 3.4 eV, which enables optical absorption in the UV range (Figure 5d). Importantly, these calculations were performed for relatively small molecular fragments and therefore primarily describe the local electronic structure. They do not account for collective excited states arising from extended hydrogen-bonded networks, polymer aggregation, structural disorder, or intermolecular through-space electronic coupling, all of which have been proposed to contribute to the photophysical behavior of nonconjugated polymeric materials.^46^ The intrinsic fluorescence of the cross-linked chitosan hydrogel likely originates from a combination of factors rather than a single emissive chromophore.^47,48^ Consequently, the DFT calculations should be regarded as qualitative evidence that cross-linking alters the electronic structure and enables UV absorption rather than as a direct description of the emissive states responsible for fluorescence.

Whereas pristine chitosan hydrogels exhibit pronounced fluorescence under 365 nm excitation, incorporation of PNOR results in strong emission quenching without substantial shifts of the emission maximum (Figure 5c). The presence of only a minor spectral shift indicates that the emissive states associated with the chitosan matrix remain largely unchanged. Instead, the decrease in fluorescence intensity suggests that excited-state energy is dissipated through newly introduced nonradiative pathways associated with PNOR. Such behavior is consistent with the well-established photoprotective function of melanin-like polymers, which efficiently convert absorbed photon energy into heat rather than re-emitting it as fluorescence.^49,50^ Additional contributions from increased intermolecular coupling within the reorganized hydrogel network cannot be excluded, as stronger electronic interactions between PNOR and chitosan may further facilitate nonradiative relaxation.

Together, the DFT calculations and optical spectroscopy indicate that confined PNOR electropolymerization substantially reshapes the electronic structure of the hydrogel interface. Formation of quinone-rich PNOR introduces electronic states responsible for enhanced optical absorption while simultaneously promoting efficient nonradiative excited-state relaxation, leading to fluorescence quenching. These findings demonstrate that hydrogel-confined electropolymerization not only modifies the morphology of the composite but also provides an effective route for engineering the optical properties.

### Percolation Networks of PNOR inside Chitosan and Conferred Redox Couples

Pristine chitosan is generally electrochemically inactive with only capacitive currents and no redox signals in the whole potential window.^41^ However, confined PNOR electropolymerization yields a hydrogel containing permanently immobilized yet electrochemically accessible catechol/quinone redox centers throughout the whole potential window (Figure 6a). The increase of the capacitive current is in agreement in reduced imaginary components of impedance in low-to-middle frequencies (Figure 6b). Overall, the hydrogel becomes intrinsically pseudocapacitive while preserving ionic permeability required for charge compensation.^51,52^ Gaussian-shaped redox peaks in the cathodic region indicate dominant contribution from surface-bound processes. The shift of the catechol/quinone couple to the cathodic region compared to the NOR monomer is expected based on the literature and was observed for electropolymerized PDA.^53^ This is also supported by DFT calculations showing a decrease in standard potentials for PNOR for longer chains (Table S6). An intriguing feature of the Cs3–PNOR electrode is that the catechol/quinone redox couple in the cathodic region becomes progressively better resolved at increasing scan rates, despite the expected increase in capacitive current (Figure 6c). The PNOR redox couple is only weakly resolved at 100 mV s^–1^ because it overlaps with the broad background response of the hydrated chitosan–PNOR matrix. At 10 V s^–1^, however, the redox peaks become substantially more pronounced while maintaining a peak separation below 100 mV, indicating rapid electron transfer and efficient charge compensation within the immobilized PNOR network. This behavior suggests that the electrochemical response is governed by overlapping processes with different characteristic time scales: slow hydrogel charging/relaxation contributes strongly at low scan rates, whereas the surface-confined PNOR redox process remains accessible at high scan rates. The enhanced peak-to-background contrast at high scan rates suggests that the capacitive contribution increases less rapidly than the faradaic response under these conditions, consistent with the nonideal charging behavior expected for hydrated polymer networks.^26^ The observed high-scan-rate phenomenon may be another result of the electropolymerization confinement. The hydrogel appears to immobilize the redox-active PNOR while still allowing rapid local ion motion for charge compensation.

**Figure 6:**
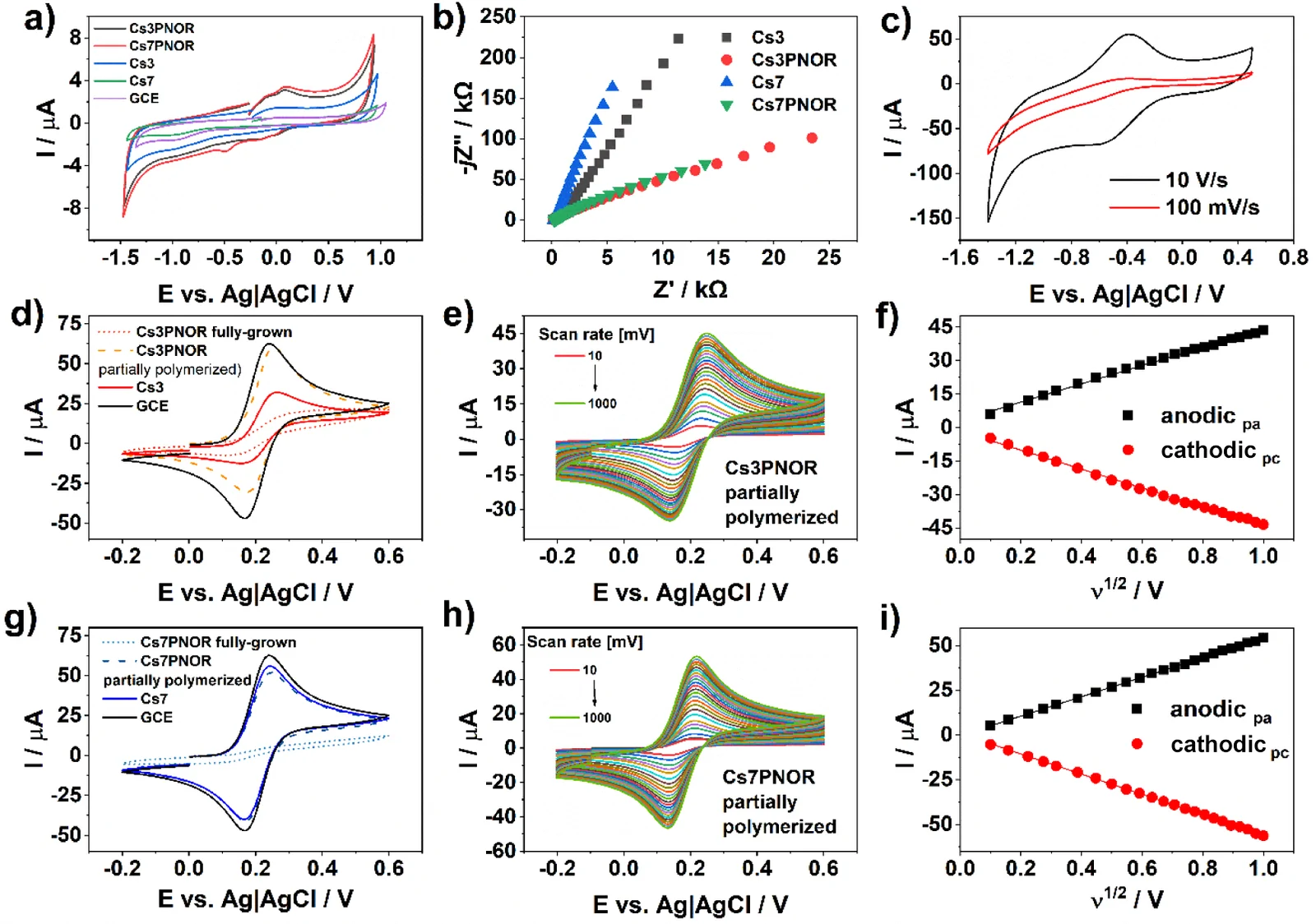
Electrochemical properties of PNOR–chitosan interfaces: a) CVs in Britton–Robbinson buffer (pH = 6), b) EIS spectra in 0.1 M PBS (pH = 7.2), c) CVs of Cs3-PNOR in 0.1 M PBS for two different scan rates, d–f) comparison between fully and partially polymerized Cs3-PNOR in the presence of 1 mM FcDM in 0.1 M KCl, g–i) analogous comparison for Cs7-PNOR.

The extent of electropolymerization can be controlled by the number of applied CV cycles during PNOR synthesis. The electrochemical results in Figure 6c–h demonstrate that the extent strongly influences conductivity and charge transport properties. Full-grown Cs-PNOR coatings, exhibit very low currents in the presence of the FcDM redox couple indicating relatively homogeneous coverage of the surface with an impermeable and/or insulating PNOR layer. In contrast, partially grown PNOR yields significantly higher redox signals, comparable to the unmodified GC substrate. Both partially polymerized Cs-PNOR systems obey the Randles–Ševčík relationship, indicating a diffusion-controlled redox process. In light of these results, one can state that electropolymerization proceeds through two electrochemically distinct regimes. During the early stages, isolated PNOR domains facilitate electron transport without blocking access of solution species to the electrode. Continued polymer growth ultimately produces a dense continuous film that limits diffusion of the redox probe and suppresses faradaic currents.^54^ At intermediate polymerization stages, the PNOR network remains sufficiently open to permit diffusion of redox species while simultaneously providing additional electronic pathways for charge transport including electron tunneling through potential barriers.^55,56^ Additionally, the presence of PNOR functional groups enables the formation of interactions, such as hydrogen bonds and π–π, which may lead to the accumulation of Fc­(MeOH)_2_ within the layer and a local increase in its concentration near the electrode surface. Collectively, the electrochemical measurements reveal that confined PNOR electropolymerization transforms chitosan from an electrochemically inert hydrogel into a permanently redox-active interface. Moreover, the degree of polymer growth determines the balance between electronic connectivity and mass transport, enabling continuous tuning of the transition from an ion-permeable redox network to a compact transport barrier.

The influence of the PNOR oxidation state was tested by applying highly anodic (+1.5 V) and highly cathodic (−1.0 V) potentials for 3 min to change the redox state of the as-formed PNOR–chitosan composite (Figure S5). Both anodic and cathodic polarizations clearly alter the system impedance, but clearly, cathodic treatment leads to a significant reduction of the system impedance in the low-frequency regime, where diffusion is typically a rate-limiting step. The optical properties are basically not altered significantly due to polarization.

It should be noted that the EIS spectrum is not recorded during the polarization at highly anodic or cathodic potentials but rather right after polarization in near-open-circuit conditions. Therefore, polarization enables a quasi-permanent increase or decrease in the system impedance at low frequencies by modulating the redox state of PNOR. Interestingly, the same pattern was preserved across three independent samples. Because of the lack of chitosan electroactivity, this behavior would be impossible without PNOR as a component. Moreover, this impedance switching property suggests potential future applications in bioelectronics as memristors.

The investigated chitosan–PNOR system is not a competitor to composites such as chitosan–PANI, chitosan–PEDOT, or chitosan–MXenes in terms of mechanical properties, bulk conductivity, and self-healing capacity.^57^ Rather, it is a redox-active hydrogel with possible applications in different types of biosensing as shown in previous work.^58,59^ Our work demonstrates that PNOR confers redox activity within the hydrogel with a novel element regarding in situ electropolymerization and its unique possibility of tailoring the hydrogel microstructure. It also fits into the category of stimuli-responsive hydrogels similar to the ones presented in this review.^60^

Finally, the advantages of the system can be condensed into these bullet points:

- PNOR is generated *in situ* by spatially localized electropolymerization rather than introduced as a preformed conductive filler.
- The resulting functionality is intrinsically redox-active: permanent catechol/quinone centers remain electrochemically accessible within the hydrogel.
- Third, the same modification gives an optical response—increased broadband absorption and fluorescence quenching without adding a separate optical component.
- Fourth, the reaction is chemically programmed by the hydrogel itself: pH-dependent protonation, noncovalent interactions, solvation, and morphology control PNOR assembly.

Considering the broad applications of chitosan hydrogels in the field of tissue engineering, it is worth noting the potential advantages of chitosan–PNOR composites in this regard.

Surface charge is a crucial parameter in regulating material–cell interaction, in particular cell attachment, mobility, proliferation, and differentiation.^61^ In our work, we show that charges of both chitosan and PNOR can be detrimental. For example, the low pH of chitosan with high abundance of R–NH_3_^+^ groups is likely to induce high density of adsorbed cells due to the attraction of negatively charged cell membranes. On the other hand, PNOR can be in the form of zwitterion, and zwitterions are known for their unique antifouling capability. It is possible that chitosan–PNOR layers could enable merging both high positive surface charge density in general with distributed protein-resistant properties.

It was shown for chitosan–genipin composite that chondrocyte cell adhesion and proliferation can also be altered due to mechanical stress–strain interactions in the hydrogel composite. We expect an analogous influence in chitosan–PNOR layers, with PNOR acting as a knob for interaction intensity and thus proliferation kinetics control. However, the interaction between chitosan and genipin seems not to be the same in nature because PNOR does not act as a cross-linking agent in the same way. Chitosan is already cross-linked by gultaraldehyde in our work, and PNOR acts as a modifier of the higher-order physical structure.^62^

Moreover, because PNOR offers a smooth surface and effectively resists biofouling , it has been shown to boost cell adhesion kinetics.^11^ It is very likely that this property is retained in chitosan–PNOR composites, but this would require experimental validation.

## Conclusions

The present study demonstrates the confined, chemically programmed electropolymerization of polynorepinephrine in the chitosan matrix. By controlling the protonation state of the hydrogel through the preparation pH, the local electrostatic environment, morphology, and hydration of the polymer network are modified, resulting in fundamentally different pathways of confined PNOR growth. The combined electrochemical, microscopic, spectroscopic, XPS, and DFT investigations reveal that the formation of the PNOR–chitosan composite is governed predominantly by hierarchical noncovalent self-assembly. DFT-COSMO calculations indicate that electrostatic interactions between protonated chitosan and zwitterionic PNOR constitute the principal driving force for composite formation, while π–cation interactions provide additional stabilization and hydrogen bonding primarily preserves the integrity of the individual polymer networks. Consistent with this interpretation, XPS reveals only minor differences in chemical composition despite pronounced changes in morphology, electrochemical response, and optical properties, demonstrating the importance of physical, mesoscopic organization. The confined electropolymerization process transforms the initially electrochemically inert hydrogel into a permanently redox-active interface containing immobilized catechol/quinone centers that remain electrochemically accessible even at scan rates as high as 10 V s^–1^. Furthermore, the results suggest that PNOR growth proceeds through distinct electrochemical regimes, evolving from isolated redox centers to an interconnected redox percolation network capable of mediating charge transport, and finally to a dense polymer layer that progressively limits mass transport. Beyond electrochemical functionality, oxidation of PNOR introduces low-energy electronic states that broaden optical absorption, while the intrinsic excited-state relaxation pathways of the melanin-like polymer efficiently quench the fluorescence of the cross-linked chitosan matrix. We anticipate that the concept of hydrogel-confined electropolymerization will be applicable beyond the PNOR–chitosan system and provide new design principles for bioinspired electroactive hydrogels.

## Supplementary Material



## Notes

Further details of experimental procedures and details of DFT simulations; Lewis structures: oxidation of NOR monomer, assumed polymerization pathways, protonation equilibria, electrostatic interactions, pi–cation interactions, hydrogen bonds (Schemes S1–S6); experimental results: optical microscopy images, water contact angle results, electrochemical polymerization at various pH, CV stability of redox couples of chitosan–PNOR system, influence of PNOR redox state on the system impedance (Figures S1–S5); computational results: DFT-COSMO charge density maps and sigma-profiles, additional LDOS spectra, molecular models of chitosan and PNOR (Figures S6–S12); and XPS peak assignment details, DFT-COSMO solvation energies, energetics of intermolecular interactions, HOMO–LUMO gaps (Tables S1–S9) (PDF)

## Notes

Conceptualization: A.S., A.O., and T.O. Methodology: A.S. and A.O. Validation: A.S., M.S., Ł.G., P.O. Investigation: A.S., A.O., M.S., Ł.G., and P.O. Formal analysis: A.S. and A.O. Data curation: A.S. Writing––original draft: A.S. and A.O. Writing––review and editing: A.S., A.O., and T.O. Visualization: A.S. and A.O. Supervision: T.O
