# Supplementary material for: Hydrogel-Controlled Electropolymerization of Polynorepinephrine through Confined Noncovalent Self-Assembly
Source: Langmuir. 2026 Oct 19;42(43):31979–91. doi: 10.1021/acs.langmuir.6c04478 (PMC13738687; doi:10.1021/acs.langmuir.6c04478)
Supplement: Supplementary file 1 [file la6c04478_si_001.pdf]

Supplementary Information File

for

Hydrogel Controlled Electropolymerization of Polynorepinephrine through Confined Non-Covalent Self-Assembly

Agata Smułka[a*], Mirosław Sawczak[b], Łukasz Gaweł[c], Piotr Okoczuk[d], Tadeusz Ossowski[a], Adrian Olejnik[e]

[a] Department of Analytical Chemistry, University of Gdańsk, Wita Stwosza 63, 80-308 Gdańsk, Poland

[b] Photophysics Department, Institute of Fluid-Flow Machinery, Polish Academy of Sciences, Fiszera 14, 80-231 Gdańsk, Poland

[c]Department of Corrosion and Electrochemistry, Faculty of Chemistry, Gdansk University of Technology, Narutowicza 11/12, 80-233 Gdansk, Poland

[d] Faculty of Applied Physics and Mathematics, Institute of Nanotechnology and Materials Engineering, Gdańsk University of Technology, Narutowicza 11/12, 80-233 Gdańsk, Poland

[e]Department of Optoelectronics, Faculty of Electronics, Telecommunications and Electronics, Gdańsk University of Technology, Narutowicza 11/12, 80-233 Gdańsk, Poland

* Corresponding author: agata.smulka@phdstud.ug.edu.pl

Supplementary experimental data:

- Materials and reagents

All reagents used were sourced from Sigma Aldrich, of analytical grade, and used as received without further purification. The compounds used are: low-molecular-weight chitosan (Cs), acetic acid, glutaraldehyde, sodium hydroxide, potassium chloride, hydrochloric acid, norepinephrine, potassium hexacyanoferrate(III) and potassium hexacyanoferrate(II), 1,1'-ferrocenedimethanol, phosphoric acid(V), boric acid, and Tris buffer. Tris buffer was diluted 10-fold with demineralized water, and its pH was then adjusted by adding HCl or NaOH. Britton-Robinson buffer was prepared from phosphoric, boric, and acetic acids, and its pH was adjusted using NaOH. Phosphate-Buffered Saline (PBS) was obtained from ThermoFisher Scientific (USA) and prepared by dissolving tablets in deionized water to achieve a concentration of 0.01 M and a pH of 7. When preparing solutions (including buffers), the pH was monitored potentiometrically using Mettler Toledo InLab Micro electrodes (Switzerland). Deionized water obtained using a Hydrolab system was used in the experiments.

- Physicochemical measurements

The surface topography maps were studied with an NTEGRA Aura (NT-MDT) atomic force microscope (AFM) setup in contact mode and using SICON probes (AppNano). The nominal geometric parameters of the probe are as follow: cantilever length 450 µm, width 49 µm, thickness 2.5 µm, tip curvature radius <10 nm. Images were taken at the surface of freshly prepared samples. A plane was subtracted from the images to recompensate for sample placement. Also, the parabola was substracted from images to compensate the piezoelectric non-linearity. The root mean square roughness R_q is given by $R_q = \sqrt{\frac{1}{N}\sum_{n=1}^{N}(z_n - \bar{z})^2}$, where $\bar{z}$ is an average height and z_n is a height at a given point. Mean roughness R_a is determined from the average of the absolute deviations from the mean height rather than the squared deviations [1].

The sample surface topography and film thickness was analyzed by a DektakXT (Bruker, Billerica, MA, USA) profilometer. The samples were prepared by depositing an appropriate volume of hydrogel onto a GCE electrode; on some of the samples, noradrenaline was electropolymerized.

The Raman analysis was performed using microRaman spectrometer (InVia, Renishaw, UK) equipped with a 532 nm excitation (Ar-ion laser) / 785 nm semiconductor laser. The laser power was reduced to 0.5 mW to avoid sample heating. Raman spectra were recorded in the range of 100–3200 cm^{-1}.

The absorbance spectra were recorded using a UV-vis spectrophotometer (Lambda 35, Perkin-Elmer) equipped with a diffuse reflectance accessory / in a transmission mode. Spectra were recorded in the range of 200 to 700 nm with a scanning speed of 60 nm/min.

The fluorescence spectra were recorded by means of a custom-built system equipped with 365 nm 500 mW UV LED used as an excitation source. Additional bandpass filter (UG11, Schott) was mounted on the source to remove residual LED radiation in the visible range. Samples were illuminated at an angle of 45 degrees. The fluorescence signal was collected with a quartz lens and analysed using a 0.3m monochromator (SR303i, Andor) equipped with 600 groves/mm grating and recorded with an ICCD detector (DH740, Andor). Additionally, in the detection path, a bandpass filter (GG400, Schott) was used for blocking the excitation wavelength.

Scanning electron microscopy (SEM) analysis were performed using an FEI Quanta 250 FEG instrument (Thermo Fisher Scientific, Waltham, MA, USA) equipped with a Schottky field emission gun operating, at an accelerating voltage of 20 kV.

X-ray Photoelectron Spectroscopy (XPS) analyses were performed using the Escalab 250Xi spectrometer (ThermoFisher Scientific) equipped with an AlKα X-ray source featuring a 250 μm spot size. A pass energy of 20 eV was applied. Low-energy electrons and Ar^+ ions were employed for charge compensation, with final peak calibration to adventitious carbon C 1s at 284.7 eV. Peak deconvolution was carried out using Avantage software.

Optical microscopy was performed using an Optica IMM-1 microscope at 20x magnification. The images were viewed using Vision Pro Plus software.

The contact angle and surface free energy were determined using a Kruss (Hamburg, Germany) DSA100 goniometer. The analysis was performed using GCE electrodes coated with a hydrogel formed by the electropolymerization of norepinephrine, which were thoroughly rinsed with water and dried. Using a syringe, 2 μL of liquid-water, formamide, and diiodomethane-was applied to the surfaces. The image was recorded using a CCD camera, and the parameters were determined using ADVANCE software. The Young-Laplace method was used to determine the average contact angle. Next, the surface free energy and its components (polar and nonpolar) were calculated using the methods of Owens, Wendt, Rabel, and Kaelble.

- Quantum chemistry simulations

All density functional theory (DFT) simulations were performed using the QuantumATK v2025.09 software package from Synposys [2]. All the molecular structures were created using the Builder tool of the ATK package.

DFT calculations were performed using norm-conserving PseudoDojo core potentials and associated basis sets with Linear Combination of Atomic Orbitals (LCAO) approach as implemented in ATK software [3]. The level of theory was generalized gradient approximation (GGA) with PBE exchange-correlation functional. Moreover, D3 dispersion correction was applied to achieve better description of the van der Waals interactions [4].

DFT-COSMO as implemented in QuantumATK [5] was used for calculating the surface charge density maps and sigma-profiles of chitosan, NOR and PNOR models. LDOS spectra were obtained with gaussian broadening (25 meV) of the energy eigenvalues. Zero on the energy scale for each spectrum was referenced against the Fermi level. Differences between Fermi level positions correspond to the differences in ionization potentials (work functions). Multipole boundary conditions for Poisson equation we applied. Geometry optimizations for molecules were performed with 0.05 eV/A threshold using Broyden–Fletcher–Goldfarb–Shanno (L-BFGS) optimizer.

Redox potentials of catechol-quinone couples were estimated according to the Norskov computational hydrogen electrode (CHE) approach [6]:

$$HO - R - OH \rightarrow HO - R = O + H^+ + e^-$$

$$E_{redox} = E_{quinone} + \frac{1}{2} E_{H_2} - E_{catechol}$$

Potentials obtained initially for pH=0 (reference potential of SHE) were recalculated for other pH values according to the Nernstian shift of 59 mV/decade. Free energies were taken from DFT-COSMO calculations.

Molecular structures mentioned in the paper:

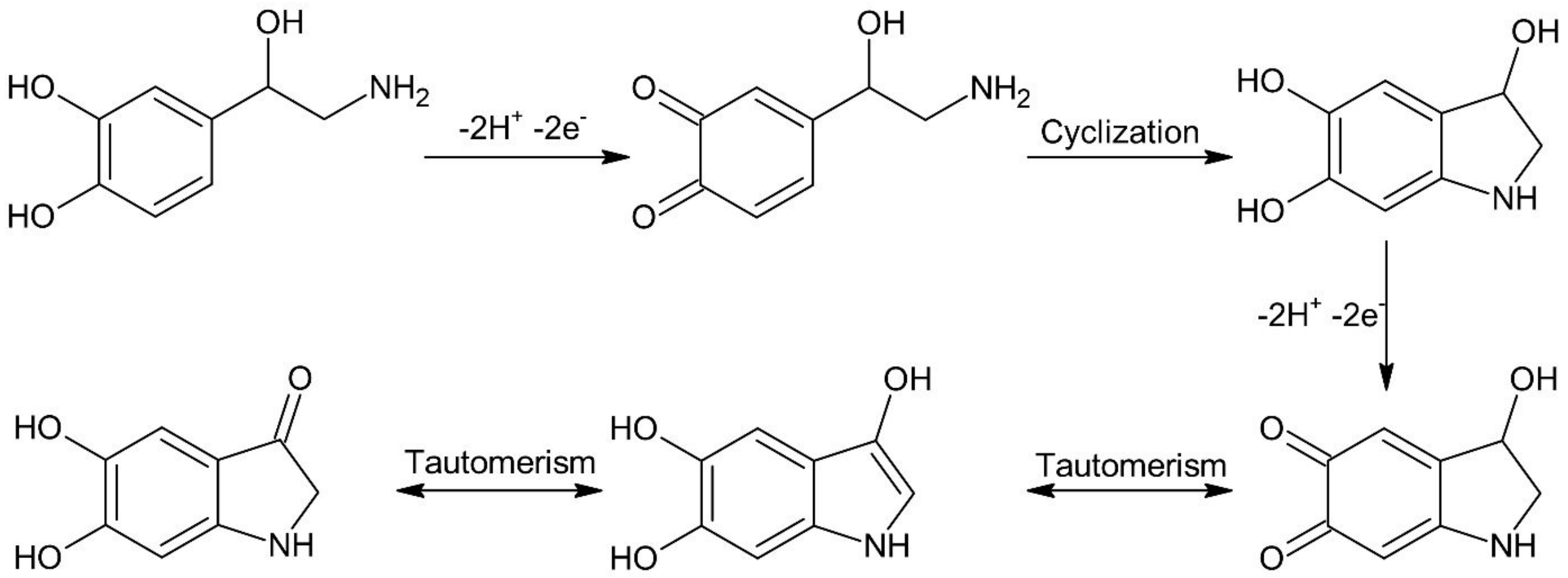

Scheme S1. NOR monomer oxidation pathways. Due to the secondary OH group, there are more possible tautomeric forms compared to the PDA

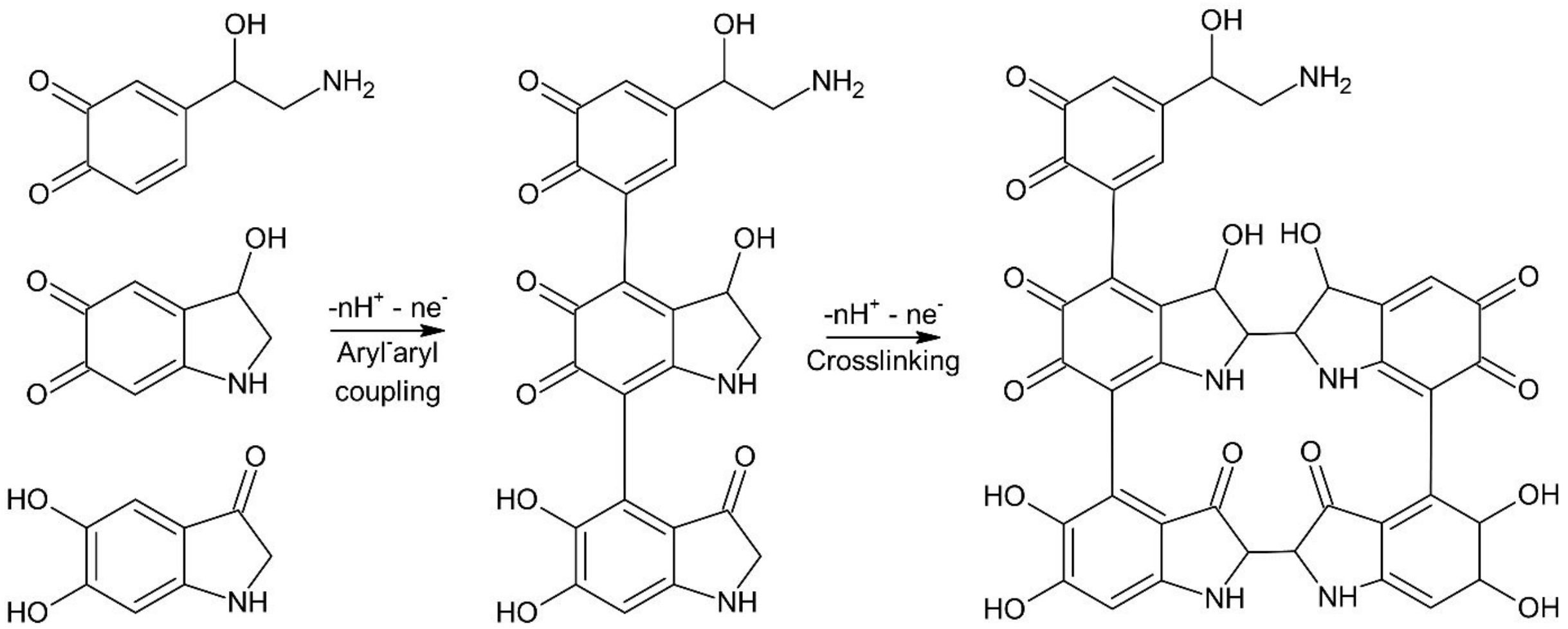

Scheme S2. Polynorepinephine polymerization pathway based on the PDA and melanin literature

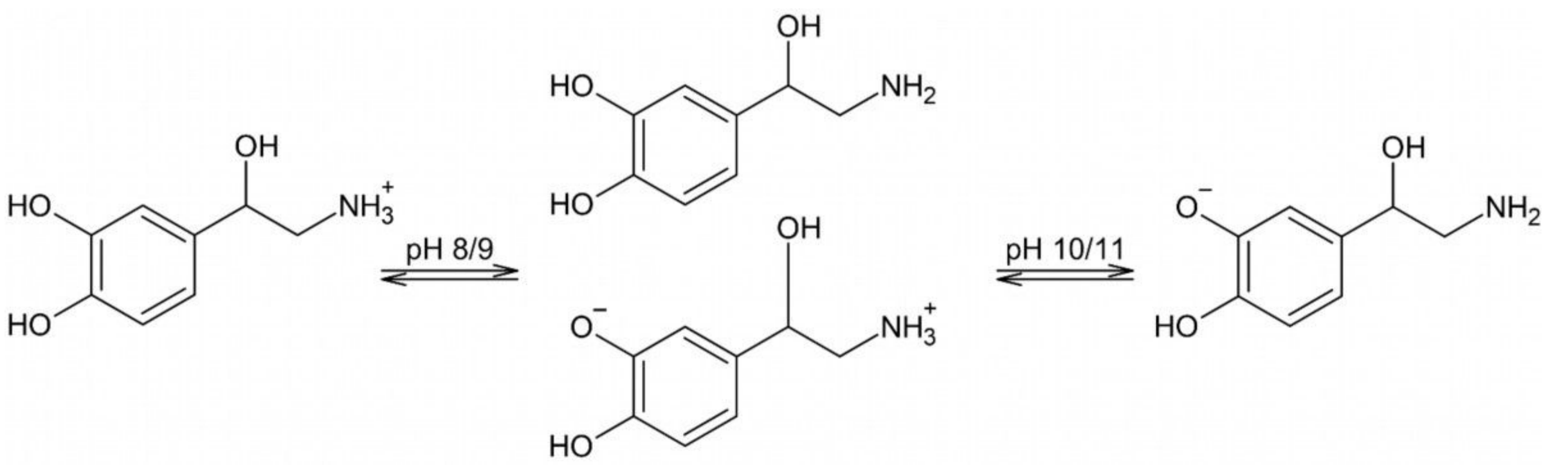

Scheme S3. Various protonation states of the NOR monomer (cationic, neutral, zwitterionic, anoinic) and associated pKa values.

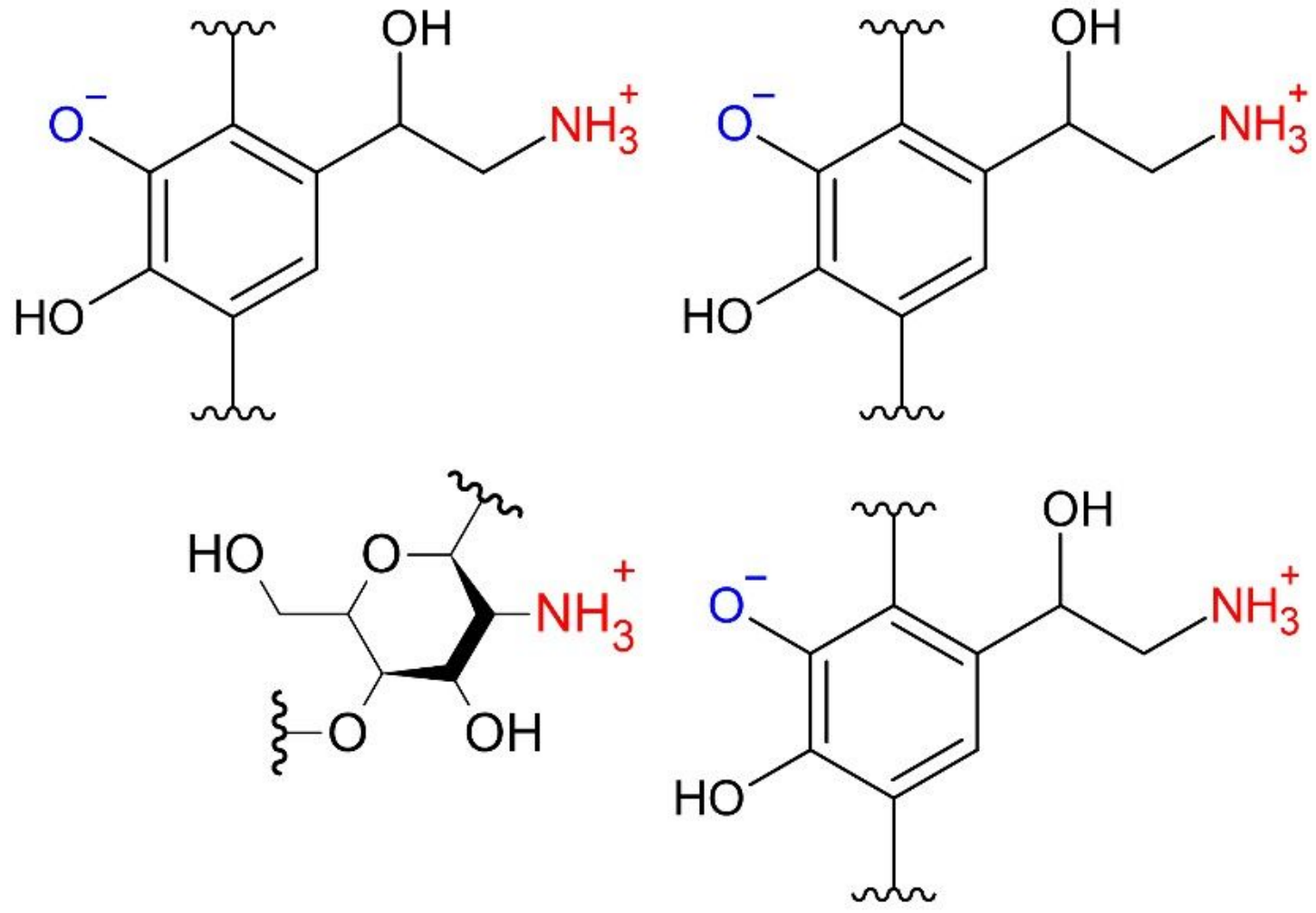

Scheme S4. Electrostatic (ionic) attractive interactions between PNOR-PNOR (exclusively in the zwitterionic form) and between chitosan-PNOR.

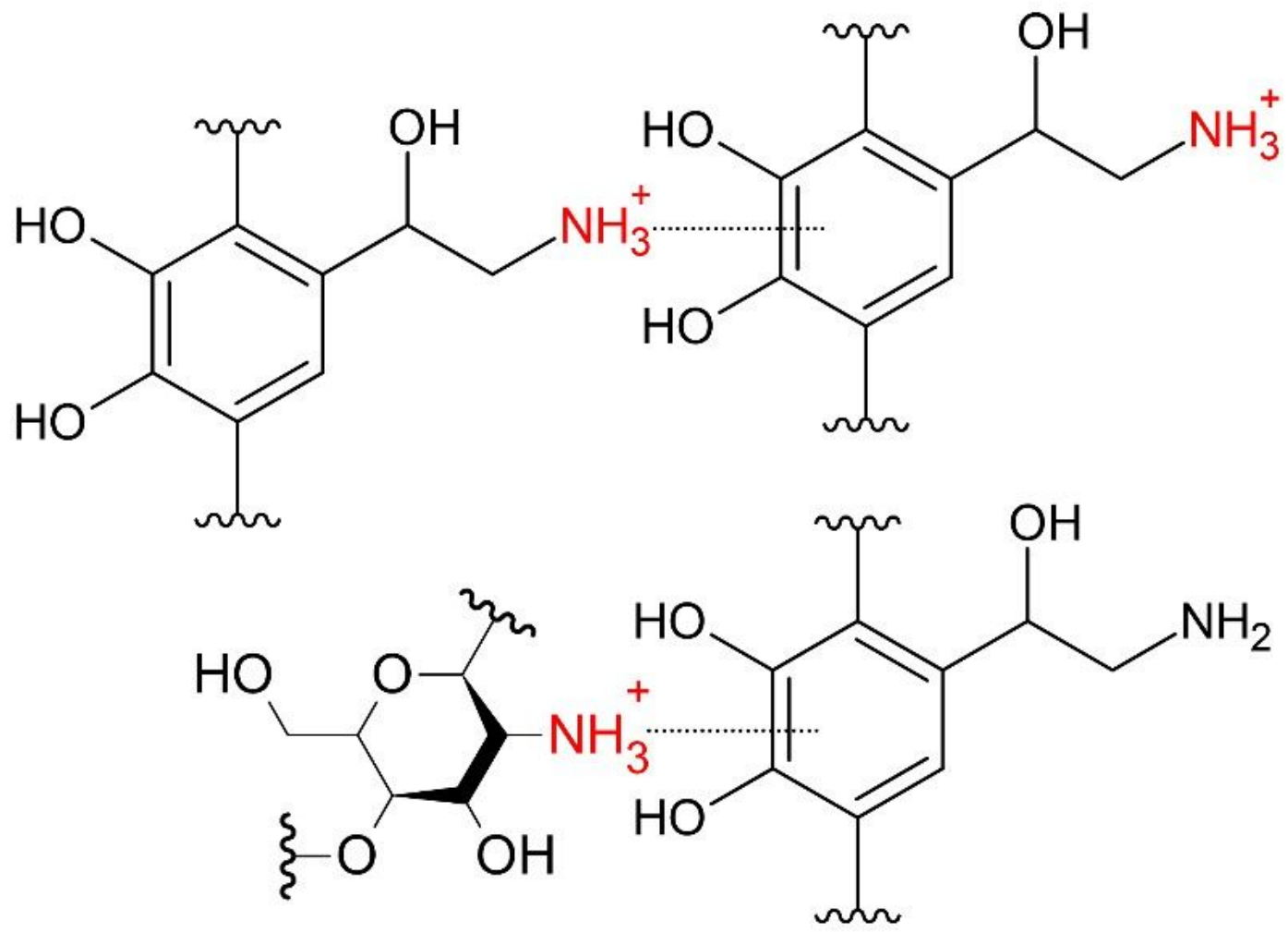

Scheme S5. π-cation interactions between PNOR-PNOR (only cationic form) and between chitosan (protonated) and PNOR (neutral or cationic forms).

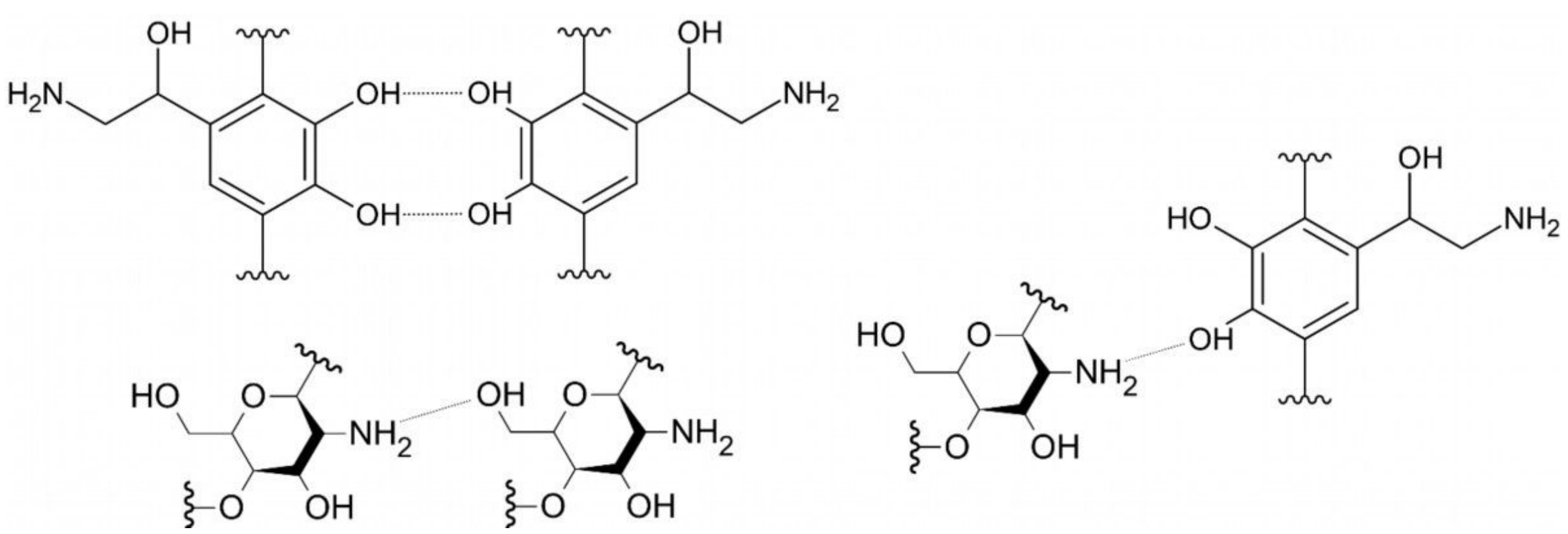

Scheme S6. Strongest hydrogen bonds formed between PNOR-PNOR, chtiosan-chitosan, and chitosan-PNOR in all neutral forms.

Supplementary experimental results:

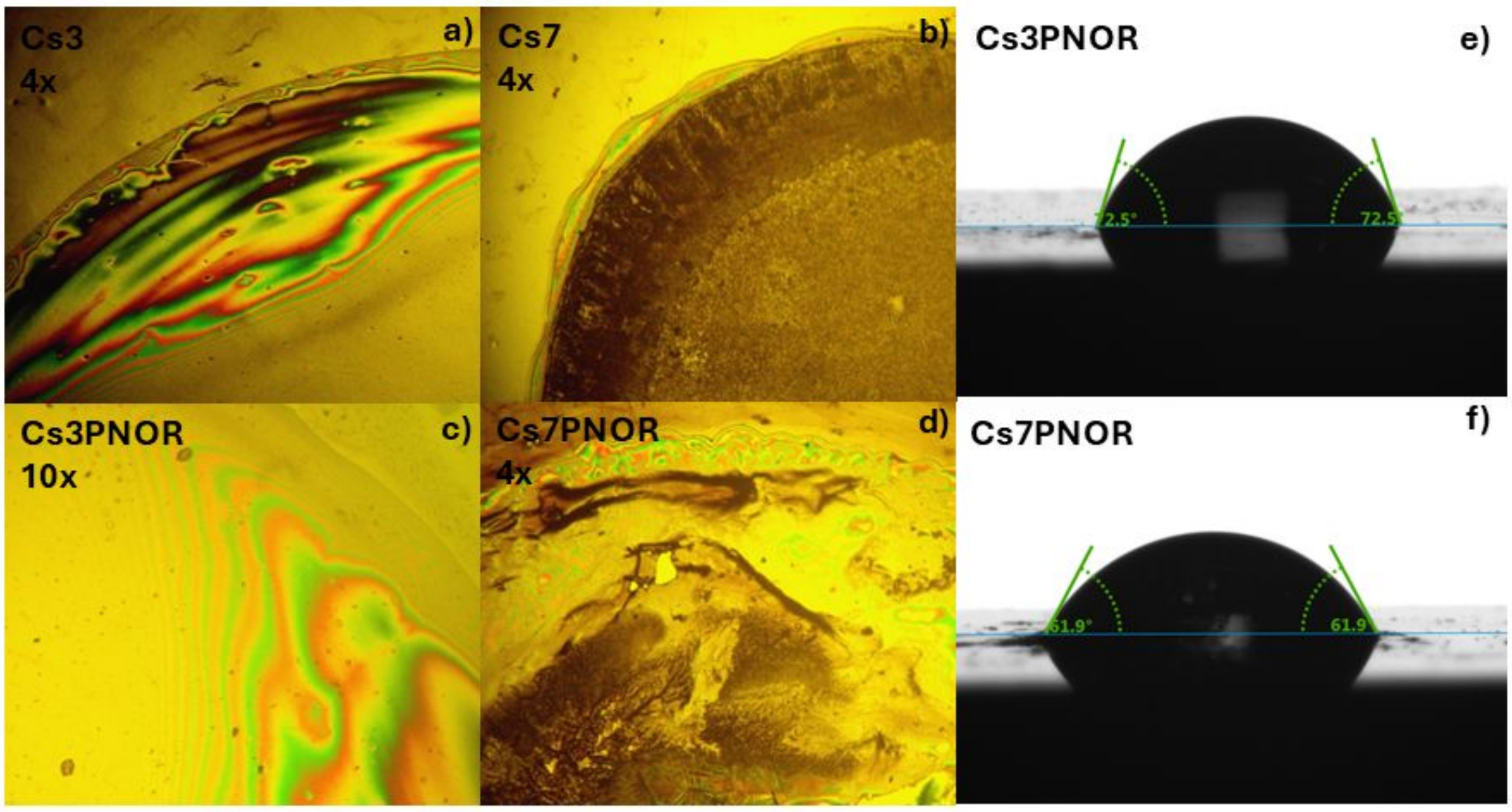

Figure S1. Optical microscopy images at 4× and 10× magnification for the a) Cs3 and b) Cs7 hydrogels and the c) Cs3PNOR and d) Cs7PNOR matrices. Water contact angle (WCA) for e) Cs3PNOR and f) Cs7PNOR matrices.

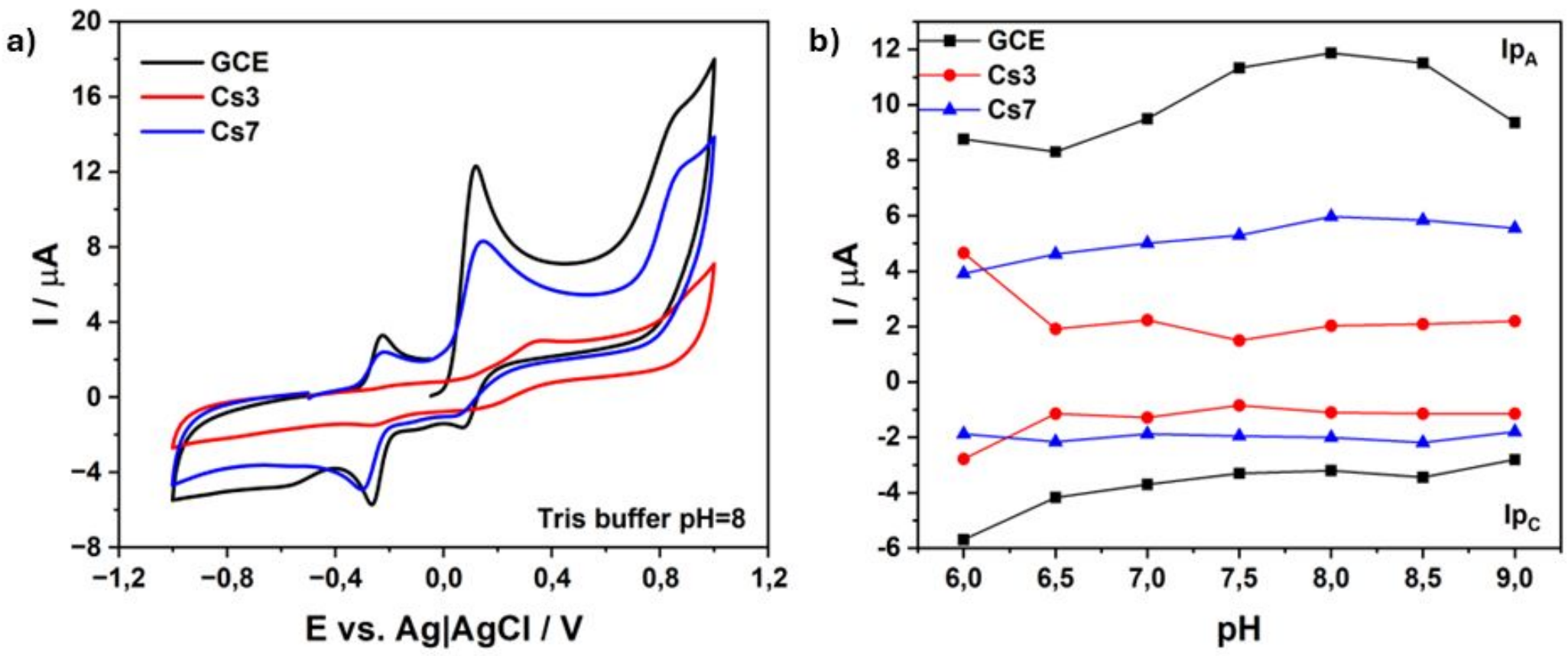

Figure S2. Comparison of the first cycles of electropolymerization: a) 1 mM polinoradrenaline in Tris buffer at pH 8 for GCE, Cs3 hydrogel, and Cs7; b) Anodic and cathodic peak currents for 1 mM polinoradrenaline for GCE, Cs3, and Cs7 in Tris buffer over a pH range of 6 to 9.

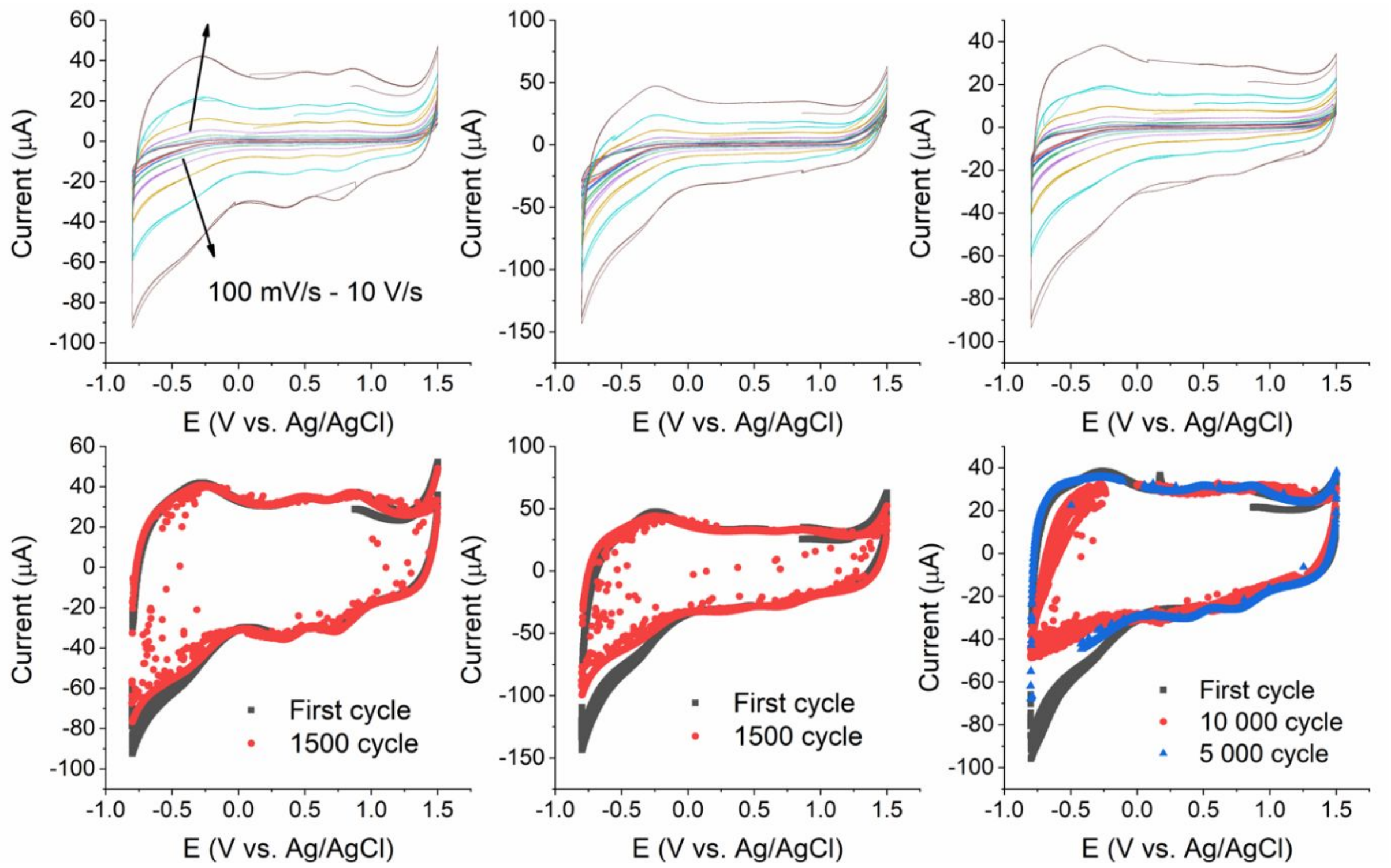

Figure S3. Repeatability and stability testing of ITO-Chitosan-PNOR electrodes; top row - CV in various scan rates; bottom row - comparison of initial and subsequent CVs after stability testing.

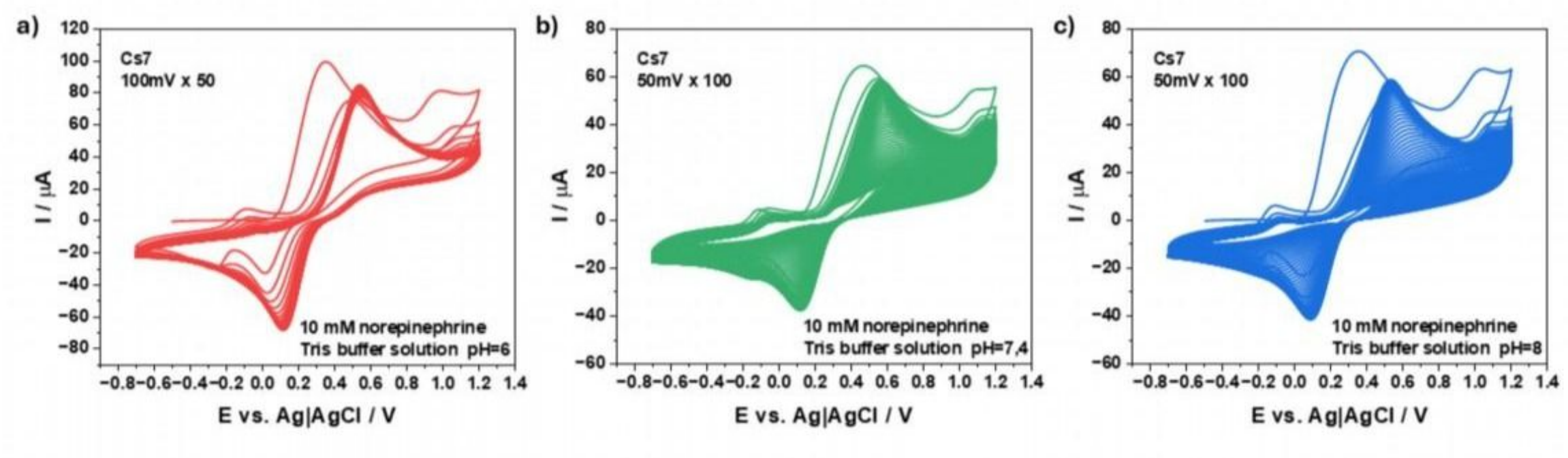

Figure S4. Comparison of NOR electropolymerization inside the Cs7 hydrogels with variable external pH of the TRIS buffer at a) pH=6; b) pH=7.4 and c) pH=8 (10 mM NOR, 50 mV/s scan rate, 100 cycles and 100 mV/s scan rate, 50 cycles).

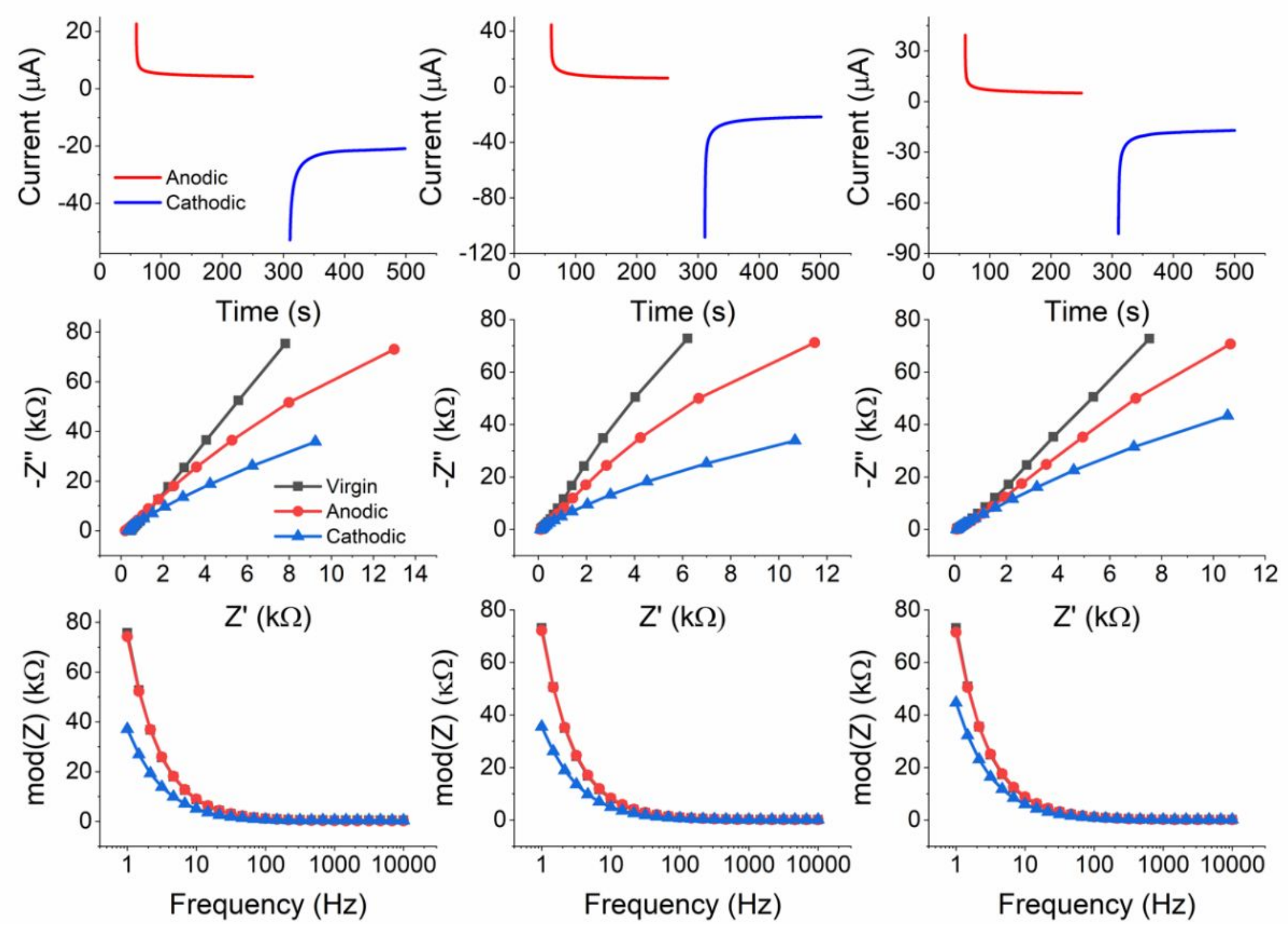

Figure S5. Influence of Chitosan-PNOR redox state on the impedance for three independent samples. Top row - chronoamperometry result of setting up anodic and cathodic state of the polymer; middle row - EIS spectra in Nyquist projection; bottom row - EIS spectra in Bode projection.

Supplementary computational results:

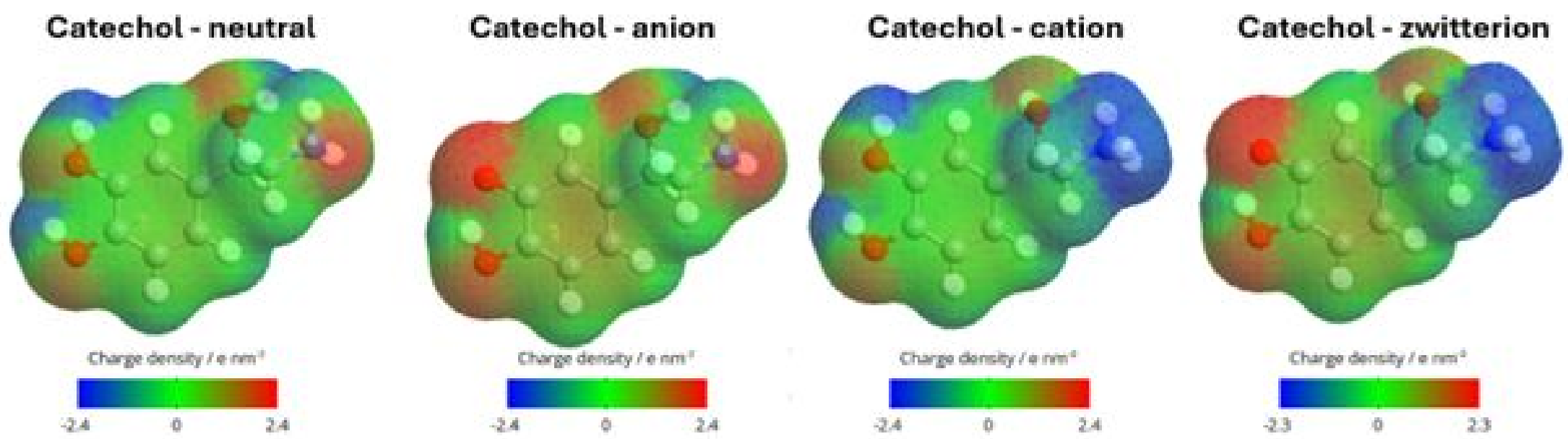

Figure S6. DFT-COSMO calculated surface charge densities for NOR monomers in different protonation states.

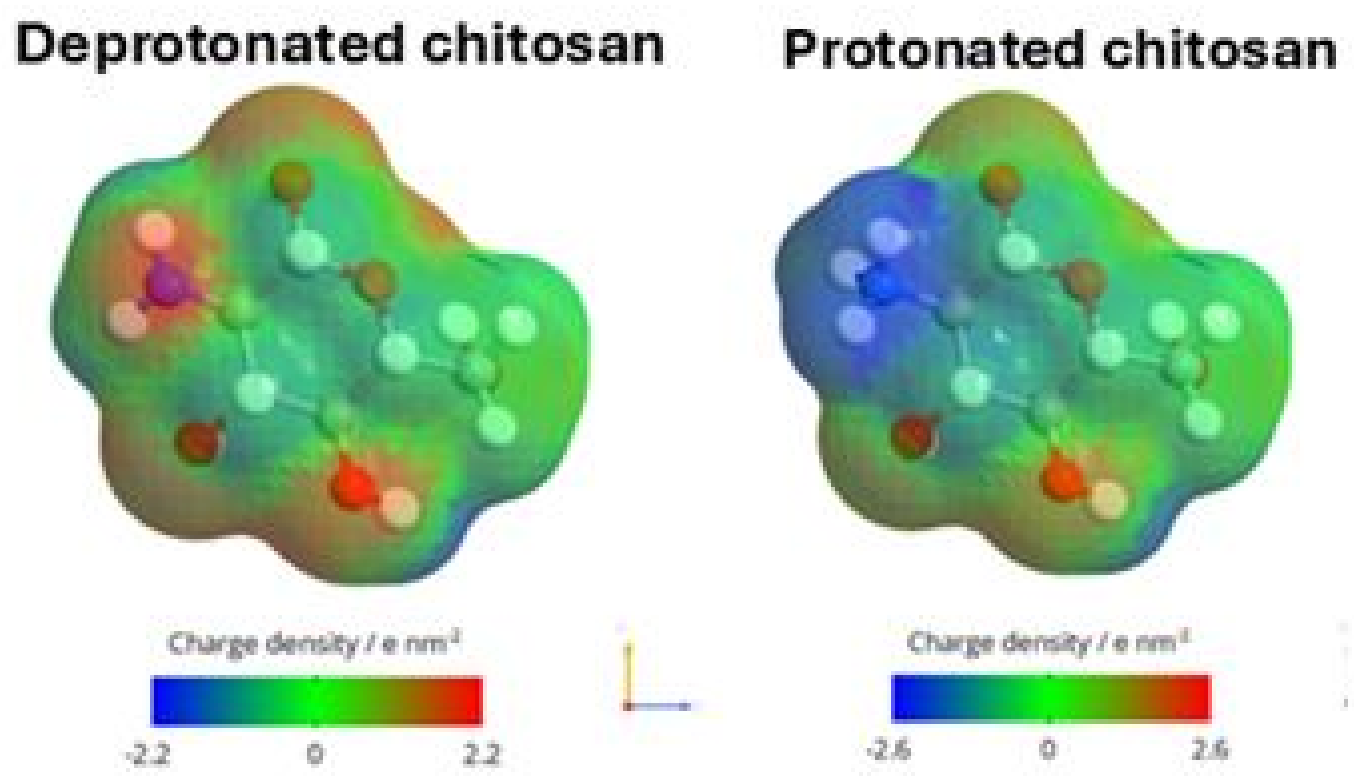

Figure S7. DFT-COSMO calculated surface charge densities for chitosan monomers in different protonation states.

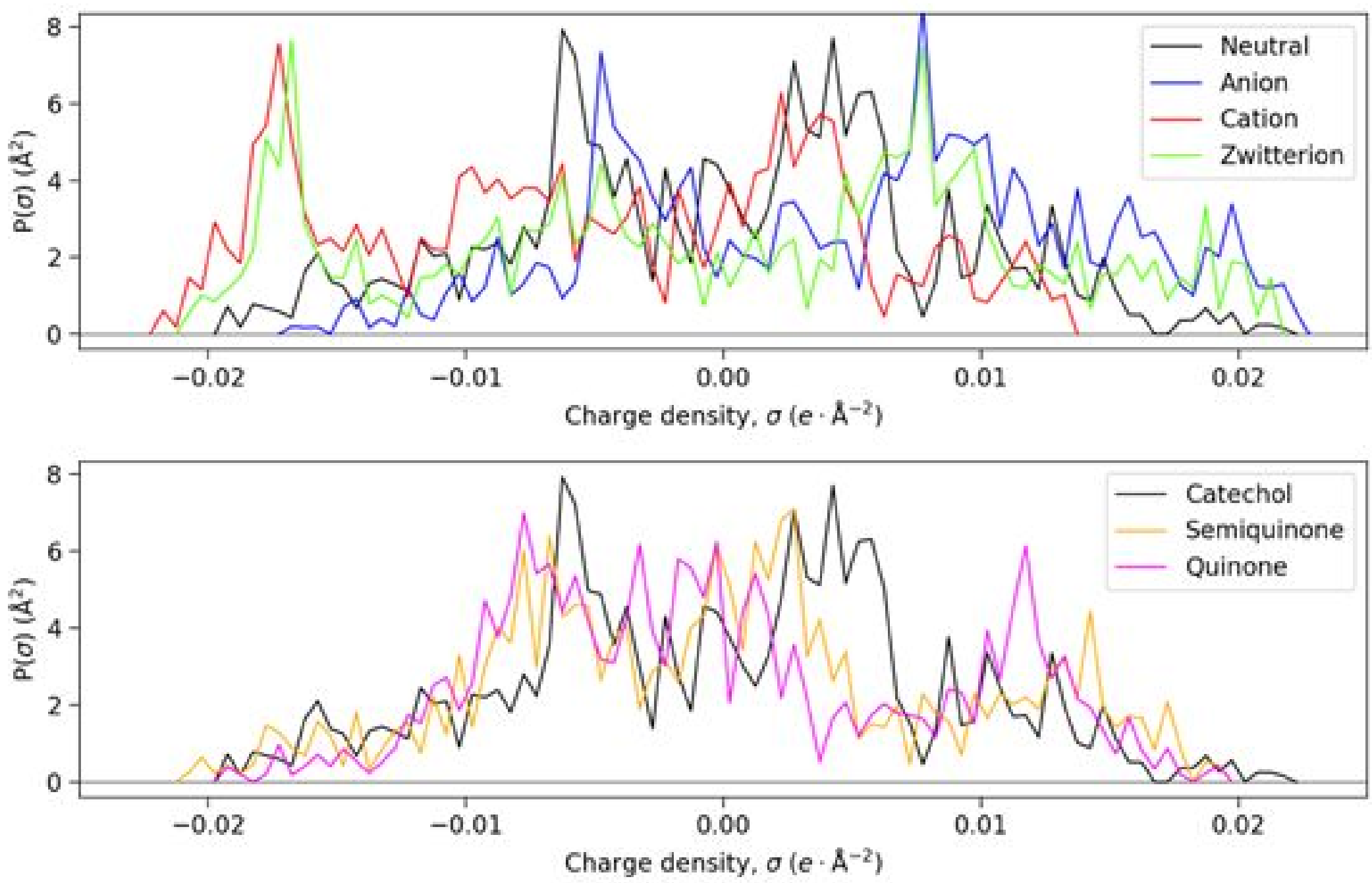

Figure S8. Sigma profiles obtained from DFT-COSMO calculations of the NOR molecule with different protonation and oxidation states (corresponding to the charge density profiles in **Figure S1)**. The most significant factor in determining the H-bond donor/acceptor character is the protonation state.

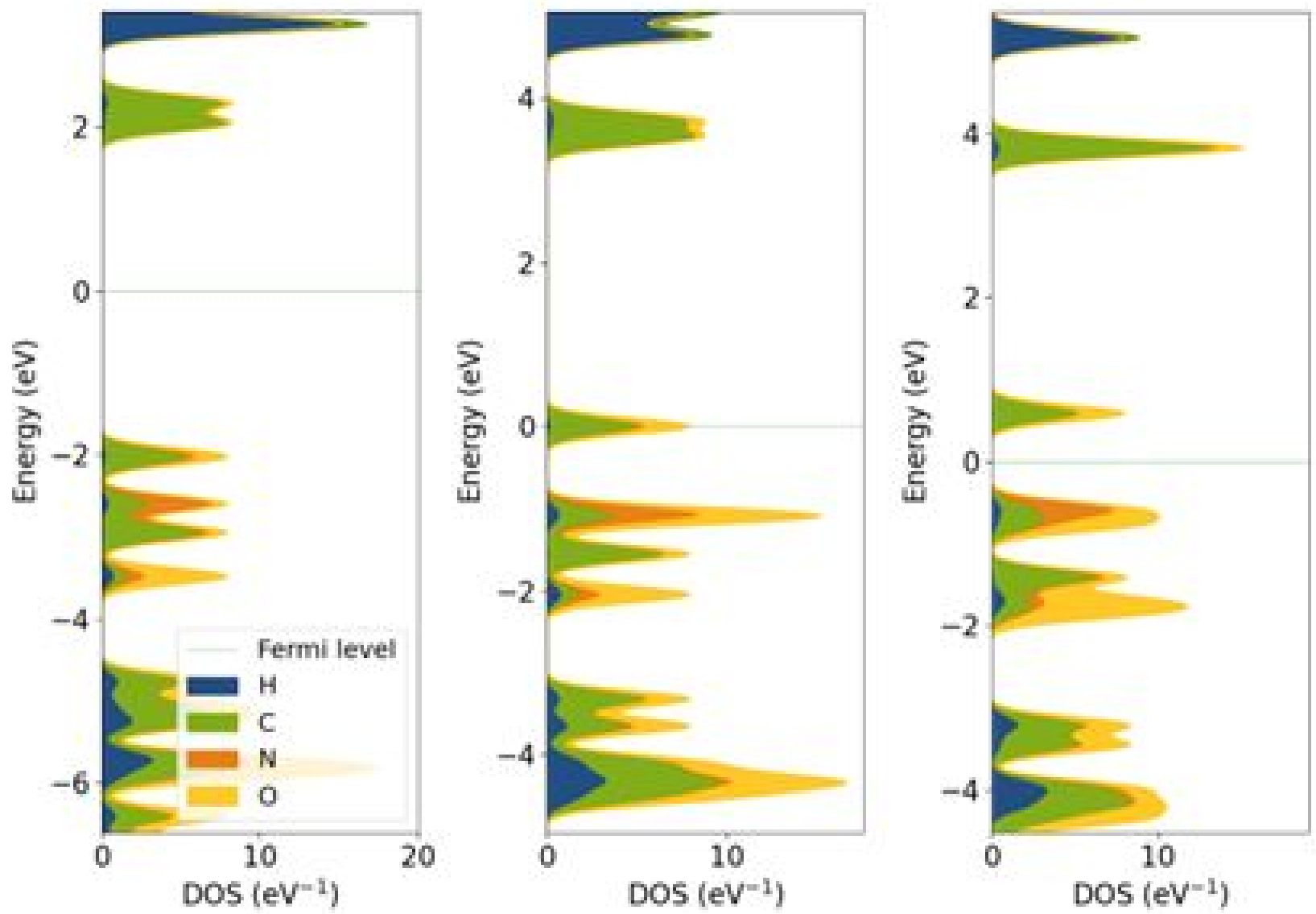

Figure S9. LDOS spectra of monomeric NOR models on different oxidation states (catechol, semiquinone, quinone). During oxidation, Fermi level drops (work function increases) and the effective bandgap changes (green is C, orange is N, yellow is O, blue is H).

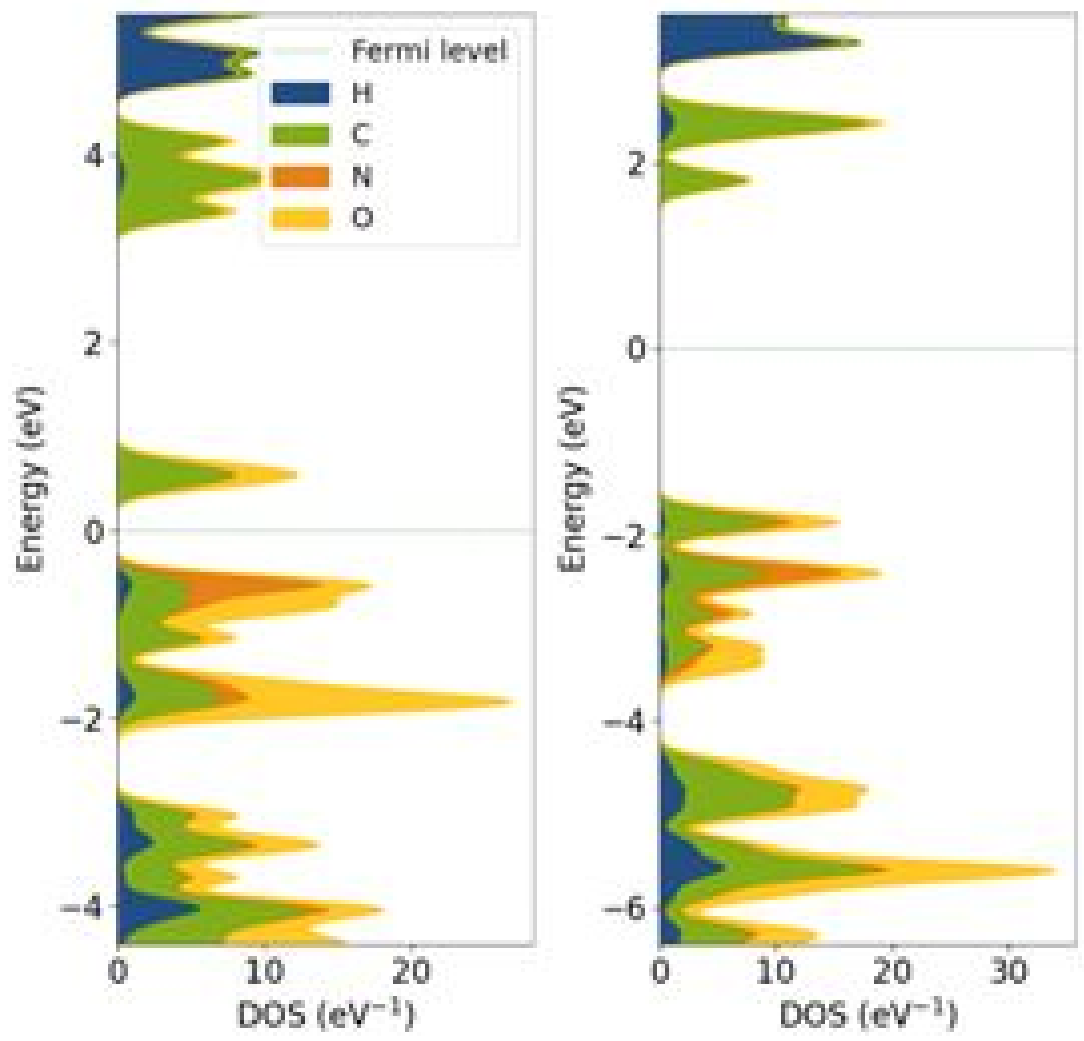

Figure S10. LDOS spectra of PNOR models: quinone-dimer and catechol-dimer. (green is C, orange is N, yellow is O, blue is H).

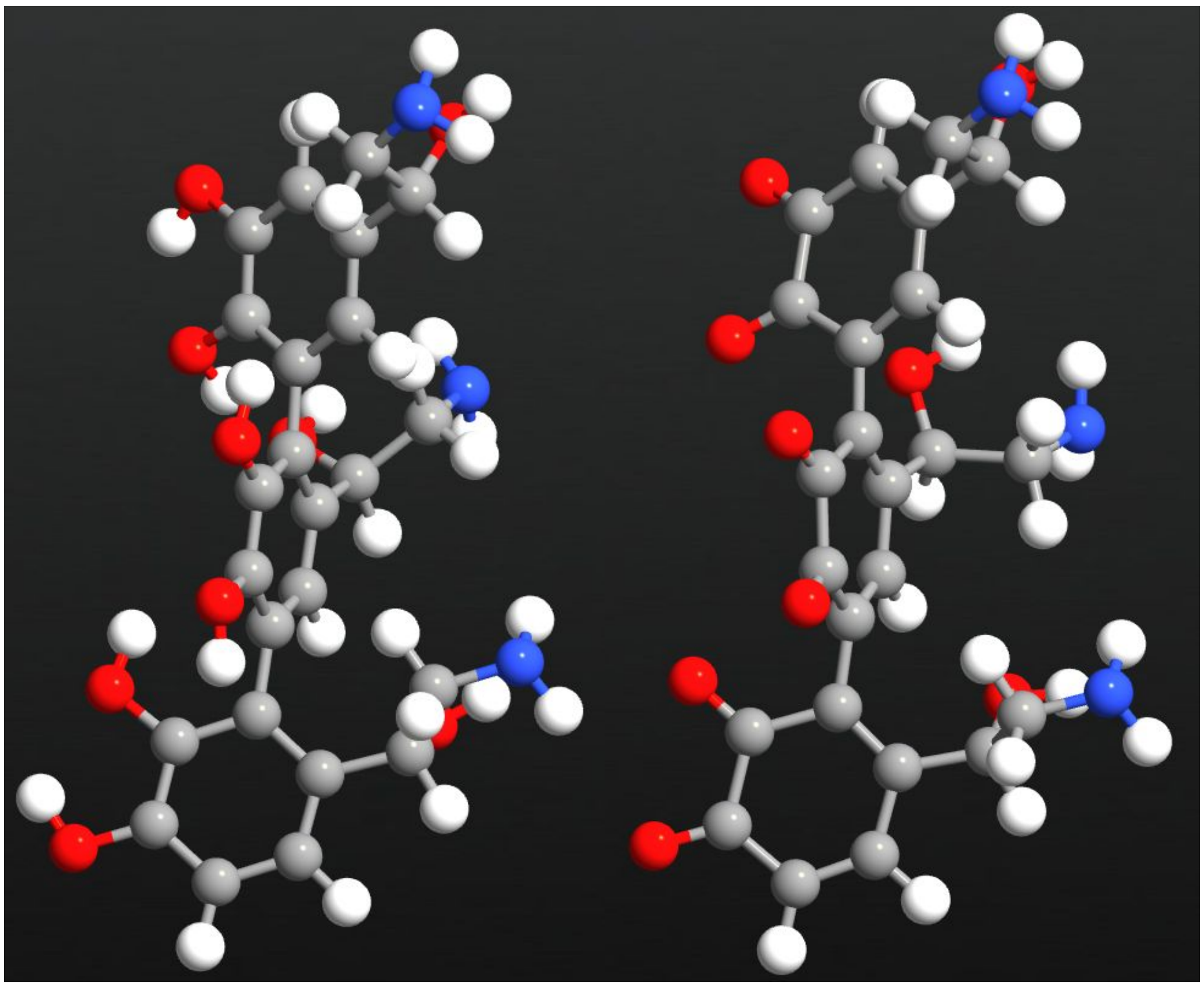

Figure S11. DFT-COSMO-optimized models of PNOR trimer in catechol and quinone forms.

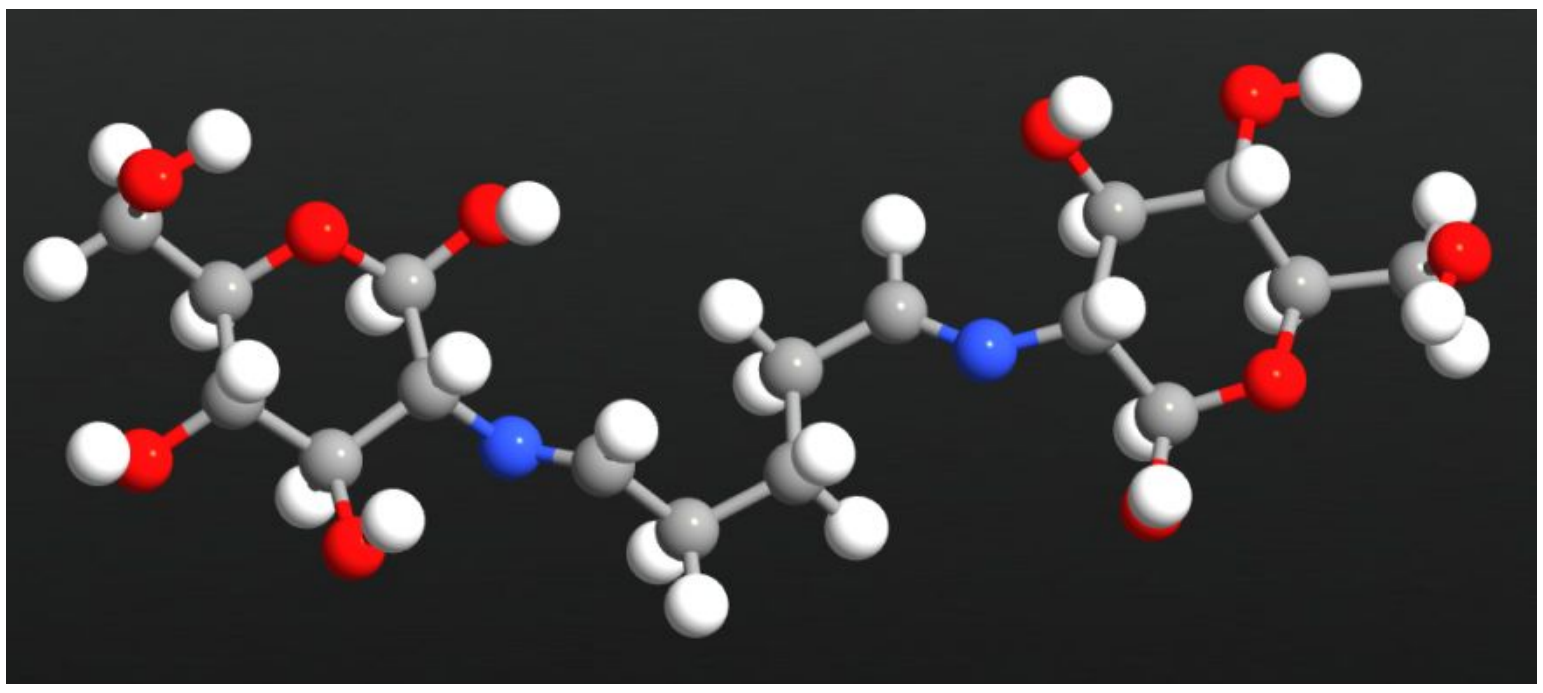

Figure S12. Simplified model of chitosan crosslinked with glutaraldehyde through formation of Schiff bases

Table S1. High-resolution XPS analysis of the hydrogel samples, performed in the binding energy range of the C 1s, O 1s, and N 1s core levels, with peak deconvolution.

	C1s				O1s		N1s		
	**C-C**	**C-O/C-N**	**C=O**	**O-C=O**	**C=O**	**C-O**	**C=N**	**C-N**	**N+/N-H**
**BE / eV**	284.7	286.1	287.9	288.5	531.5	532.9	399.1	399.8	401.4
**Cs3**	25.6	31.6	5.7	4.3	5.1	22.2	2.6	1.9	0.9
**Cs3 PNOR**	30.7	24.4	5.9	4.2	2.6	27.5	0.8	1.3	2.6
**Cs7**	27.7	23.3	6.1	6.5	13.7	19.2	1.9	1.3	0.4

**Cs7 PNOR**	34.5	24.9	4.0	4.7	5.3	22.5	0.8	0.7	2.8

Table S2. DFT-COSMO solvation energies for NOR monomer model on different protonation and oxidation states. The presence of charge (in anionic, cationic, and zwitterionic forms) have major influence of solvation, while oxidation state is less relevant.

**Model description**	**Solvation energy (eV)**
Catechol – neutral	-0,47
Catechol – anion	-2,53
Catechol – cation	-2,67
Catechol – zwitterion	-2,06
Quinone – neutral	-0,67
Semiquinone – neutral	-0,71

Table S3. DFT-COSMO solvation energies for catecholic PNOR with increasing number of mers in the chain connected by the C-C aryl-aryl coupling. Decreasing trend of solubility is observed with increasing polymer length.

Model description	**Solvation energy per monomer (eV)**
Catechol – monomer	**-0,47**
Catechol – dimer	**-0,44**
Catechol – trimer	**-0,38**

Table S4. HOMO-LUMO gaps and work functions (ionization potentials) for NOR monomer model on different protonation and oxidation states.

**Model description**	**HOMO-LUMO gap [eV]**	**Work Function (ionization potentials) [eV]**
Catechol – neutral	4,06	3,39
Catechol – anion	3,64	4,48
Catechol – cation	3,67	1,64
Catechol – zwitterion	3,04	2,75
Quinone – neutral	1,70	5,48
Semiquinone – neutral	3,51	5,06

Table S5. HOMO-LUMO gaps and work functions (ionization potentials) for PNOR models with variable chain length and oxidation states

**Model description**	**HOMO-LUMO gap [eV]**	**Work Function (ionization potentials) [eV]**
Catechol – monomer	4,06	3,39
Quinone – monomer	1,70	5,48
Catechol – dimer	3,64	3,60
Quinone – dimer	1,06	5,49
Catechol – trimer	3,43	3,87
Quinone – trimer	0,83	5,53

Table S6. Standard redox potentials for catechol/quinone redox couple for PNOR models with varying chain length obtained from computational hydrogen electrode (CHE) approach

Chain length	Catechol/quinone standard redox potential at pH=0 (as obtained from CHE)	Catechol/quinone standard redox potential at pH=7 (Nerstian-shifted)
Monomer	+1.39 V	+0.86 V
Dimer	+1.32 V	+0.79 V
Trimer	+1.27 V	+0.74 V

Table S7. Energies of NOR-NOR intermolecular interactions via hydrogen bonds (H-bonds) in neutral formal charge forms

Type of H-bond	Energy (eV)
Catechol---catechol	-0.264
Catechol---amine	-0.169
Amine---amine	-0.184

Table S8. Energies of chitosan-chitosan intermolecular interactions via hydrogen bonds (H-bonds) in neutral formal charge forms

Type of H-bond	Energy [eV]
Amine-hydroxyl	-0,250
Amine-hydroxyl	-0,167
Hydroxyl-hydroxyl	Range between -0.16 to –0.26 eV<br>Strongly dependent on the geometry

Table S9. Energies of NOR-chitosan intermolecular interactions via hydrogen bonds (H-bonds) in neutral formal charge forms

Type of H-bond	Energy [eV]
Chitosan amine --- NOR catechol	-0,158
Chitosan amine --- NOR amine	-0,033

References:

[1] Chapter 4. Data Processing and Analysis, in: Statistical Analysis, n.d. https://gwyddion.net/documentation/user-guide-en/statistical-analysis.html.

[2] S. Smidstrup, T. Markussen, P. Vancraeyveld, J. Wellendorff, J. Schneider, T. Gunst, B. Verstichel, D. Stradi, P.A. Khomyakov, U.G. Vej-Hansen, M.-E. Lee, S.T. Chill, F. Rasmussen, G. Penazzi, F. Corsetti, A. Ojanperä, K. Jensen, M.L.N. Palsgaard, U. Martinez, A. Blom, M. Brandbyge, K. Stokbro, QuantumATK: an integrated platform of electronic and atomic-scale modelling tools, J. Phys.: Condens. Matter 32 (2019) 015901. https://doi.org/10.1088/1361-648X/ab4007.

[3] M.J. van Setten, M. Giantomassi, E. Bousquet, M.J. Verstraete, D.R. Hamann, X. Gonze, G.-M. Rignanese, The PseudoDojo: Training and grading a 85 element optimized norm-conserving pseudopotential table, Computer Physics Communications 226 (2018) 39–54. https://doi.org/10.1016/j.cpc.2018.01.012.

[4] A. Smułka, A. Olejnik, D. Zarzeczańska, A. Wcisło, J. Ryl, A. Zieliński, T. Ossowski, pH-mediated charge tuning of chitosan membrane: a smart approach to control electrochemical selectivity, Electrochimica Acta 533 (2025) 146541. https://doi.org/10.1016/j.electacta.2025.146541.

[5] A. Klamt, The COSMO and COSMO-RS solvation models, WIREs Computational Molecular Science 8 (2018) e1338. https://doi.org/10.1002/wcms.1338.

[6] E. Skúlason, V. Tripkovic, M.E. Björketun, S. Gudmundsdóttir, G. Karlberg, J. Rossmeisl, T. Bligaard, H. Jónsson, J.K. Nørskov, Modeling the Electrochemical Hydrogen Oxidation and Evolution Reactions on the Basis of Density Functional Theory Calculations, J. Phys. Chem. C 114 (2010) 18182–18197. https://doi.org/10.1021/jp1048887.
